# Supplementary material for: Outcome domains and outcome measures used in studies assessing the effectiveness of interventions to manage non-respiratory sleep disturbances in children with neurodisabilities: a systematic review
Source: BMJ Open. 2019 Jun 19;9(6):e027205. doi: 10.1136/bmjopen-2018-027205 (PMC6589007; doi:10.1136/bmjopen-2018-027205)
Supplement: Supplementary data [file bmjopen-2018-027205supp001.pdf]

SEARCH STRATEGY

Applied Social Science Abstracts & Indexes (ASSIA) Via ProQuest

((((SU.EXACT("Sleep disorders") OR SU.EXACT("Sleep problems")) OR SU.EXACT("Narcolepsy")) OR (("bed time*" NEAR/3 (dysfunction* OR disorder* OR difficult* OR disrupt* OR disturb* OR delay* OR problem*)) OR (bedtime* NEAR/3 (dysfunction* OR disorder* OR difficult* OR disrupt* OR disturb* OR delay* OR problem*)) OR ("bed time*" NEAR/3 (initial* OR pattern* OR routine* OR practice* OR maintain* OR intervention* OR schedule*)) OR ("bed time*" NEAR/3 (initial* OR pattern* OR routine* OR practice* OR maintain* OR intervention* OR schedule*))) OR ((night* NEAR/3 (dysfunction* OR disorder* OR difficult* OR disrupt* OR disturb* OR delay* OR problem*)) OR (night* NEAR/3 (settle* OR settling OR wake* OR awake OR wakeful* OR waking* OR awaking* OR awakening* OR wakening*)) OR (nocturnal NEAR/3 (dysfunction* OR disorder* OR difficult* OR disrupt* OR disturb* OR delay* OR problem*)) OR (nocturnal NEAR/3 (settle* OR settling OR wake* OR awake OR wakeful* OR waking* OR awaking* OR awakening* OR wakening*))) OR ((sleep* NEAR/3 (dysfunction* OR disorder* OR difficult* OR disrupt* OR disturb* OR delay* OR problem*)) OR (sleep* NEAR/3 (settle* OR settling OR wake* OR awake OR wakeful* OR waking* OR awaking* OR awakening* OR wakening*)) OR (sleep* NEAR/3 (initial* OR pattern* OR routine* OR practice* OR maintain* OR intervention* OR schedule*)) OR (sleepless* OR insomnia* OR parasomnia* OR "night terror*" OR nightterror* OR "night mare*" OR nightmare*) OR ("sleepwalk*" OR "nighthawk*" .) OR (sleepwalk* OR sleepwalk* OR "sleep walk*" OR somnambulism) OR (narcolepsy OR "nocturnal hyperkinesia"))) AND ((SU.EXACT("Children") OR SU.EXACT("adolescentce") OR SU.EXACT("Infants")) OR (adolescent* OR baby OR babies OR child OR children OR boy OR boys OR girl OR girls OR infant* OR infancy* OR juvenile* OR paediatric OR pediatric OR preschooler* OR schoolboy* OR schoolgirl* OR schoolchild* OR teens OR teenager* OR toddler* OR youth OR youths OR "young people" OR "young person*"))) AND (((SU.EXACT("Developmentally disabled children") OR SU.EXACT("Developmentally delayed children")) OR (SU.EXACT("Developmental delays") OR SU.EXACT("Developmental disorders")) OR SU.EXACT("Angelman syndrome") OR (SU.EXACT("Attention deficit disorder") OR SU.EXACT("Attention deficit hyperactivity disorder")) OR SU.EXACT("Conduct disorders") OR SU.EXACT("Complex partial seizure disorder" OR "Epilepsy" OR "Idiopathic childhood epilepsy" OR "Landau-Kleffner syndrome" OR "Panayiotopoulos syndrome" OR "Temporal lobe epilepsy") OR

SU.EXACT("Cerebral palsy") OR SU.EXACT("Down's syndrome") OR SU.EXACT("Fragile X syndrome") OR (SU.EXACT("Prader - Willi syndrome") OR SU.EXACT("Prader-Willi syndrome"))) OR (SU.EXACT("Rett syndrome") OR SU.EXACT("Smith-Magenis syndrome") OR (SU.EXACT("Williams-Beuren syndrome") OR SU.EXACT("Williams' syndrome")) OR (ADHD or "attention deficit" OR "angelman syndrome") OR (autism or autistic or asperges* OR "cerebral palsy") OR ("conduct disorder*" OR epilepsy or epileptic) OR ("Down* syndrome" OR "Fragile x syndrome") OR ("Prader Willi Syndrome" OR "Prader-Willi Syndrome") OR ("Rett syndrome" OR "Williams syndrome")) OR (developmental NEAR/2 (disability* OR delay*)) OR neurodisability* OR (neurodevelopment* NEAR/3 (delay* OR disability* OR disease* OR disorder* OR dysfunction)) OR (neuromotor* NEAR/3 (delay* OR disability* OR disease* OR disorder* OR dysfunction)) OR (neuropsychiatr* NEAR/3 (delay* OR disability* OR disease* OR disorder* OR dysfunction)) OR (neuropsychol* NEAR/3 (delay* OR disability* OR disease* OR disorder* OR dysfunction)))
