# Supplementary material for: Outcome domains and outcome measures used in studies assessing the effectiveness of interventions to manage non-respiratory sleep disturbances in children with neurodisabilities: a systematic review
Source: BMJ Open. 2019 Jun 19;9(6):e027205. doi: 10.1136/bmjopen-2018-027205 (PMC6589007; doi:10.1136/bmjopen-2018-027205)
Supplement: Author's manuscript [file bmjopen-2018-027205.draft_revisions.pdf]

BMJ Open is committed to open peer review. As part of this commitment we make the peer review history of every article we publish publicly available.

When an article is published we post the peer reviewers' comments and the authors' responses online. We also post the versions of the paper that were used during peer review. These are the versions that the peer review comments apply to.

The versions of the paper that follow are the versions that were submitted during the peer review process. They are not the versions of record or the final published versions. They should not be cited or distributed as the published version of this manuscript.

BMJ Open is an open access journal and the full, final, typeset and author-corrected version of record of the manuscript is available on our site with no access controls, subscription charges or pay-per-view fees (http://bmjopen.bmj.com).

If you have any questions on BMJ Open's open peer review process please email info.bmjopen@bmj.com

BMJ Open

Outcome domains and outcome measures used in studies assessing the effectiveness of interventions to manage non-respiratory sleep disturbances in children with neurodisabilities: a systematic review

Journal:	*BMJ Open*
Manuscript ID	bmjopen-2018-027205
Article Type:	Research
Date Submitted by the Author:	10-Oct-2018
Complete List of Authors:	McDaid, Catriona; University of York, York Trials Unit<br>Parker, Adwoa; University of York, York Trials Unit, Department of Health Sciences<br>Scantlebury, Arabella; Newcastle University, Institute of Health and Society<br>Fairhurst, Caroline; University of York, Health Sciences<br>Dawson, Vicky; The Children's Sleep Charity<br>Elphick, Heather; Sheffield Children's NHS Foundation Trust, Department of Respiratory Medicine<br>Hewitt, Catherine; The University of York, Health Sciences<br>Spiers, Gemma; Newcastle University,<br>Thomas, Megan; Blackpool Teaching Hospitals NHS Foundation Trust<br>Beresford, Bryony; University of York, Social Policy Research Unit
Keywords:	Developmental neurology & neurodisability < PAEDIATRICS, SLEEP MEDICINE, Clinical trials < THERAPEUTICS

SCHOLARONE™
Manuscripts

For peer review only

Outcome domains and outcome measures used in studies assessing the effectiveness of interventions to manage non-respiratory sleep disturbances in children with neurodisabilities: a systematic review

Catriona McDaid[1], Adwoa Parker[1], Arabella Scantlebury[2], Caroline Fairhurst[1], Vicky Dawson[3], Heather Elphick[4], Catherine Hewitt[1], Gemma Spiers,[2] Megan Thomas[5], Bryony Beresford[6]

[1] York Trials Unit, Department of Health Sciences, University of York, York, UK

[2] Institute for Health and Society, Baddiley-Clark Building, Newcastle Upon-Tyne, UK

[3] The Children's Sleep Charity, Kale Lode, Woodfield Park, Tickhill Road, Doncaster, UK

[4] Sheffield Children's NHS Foundation Trust, Sheffield, UK

[5] Blackpool Teaching Hospitals NHS Foundation Trust, Blackpool, UK

[6]Social Policy Research Unit, University of York, York, UK

For peer review only

ABSTRACT

Objectives: To assess whether a core outcome set is required for studies evaluating the effectiveness of interventions for non-respiratory sleep disturbances in children with neurodisabilities.

Design: Systematic review

Participants: Children (≤ 18 years old) with a neurodisability experiencing non-respiratory sleep disturbance.

Intervention: Pharmacological or non-pharmacological interventions.

Outcomes

Primary: child and parent sleep-related outcomes; measures of perceived parenting confidence, efficacy or understanding of sleep management.

Secondary: child-related quality of life, daytime behaviour and cognition; parent/carer outcomes; adverse events.

Results: Thirty-nine studies (13 melatonin and 26 non-pharmacological) assessed five core outcome areas: child sleep, other child outcomes, parent outcomes, adverse events and process measures. There were 54 different measures of child related sleep across five domains: global measures; sleep initiation; maintenance; scheduling; and other outcomes. The most commonly reported measure in melatonin studies was total sleep time (n=12; 92%); and for non-pharmacological studies was the parent-reported Child Sleep Habits Questionnaire (CSHQ; 58%), both classified as global measures. Fifteen non-pharmacological (58%) and four pharmacological studies (31%) reported child outcomes other than sleep. The domains assessed (using 29 different measures) were child behaviour, quality of life, ADHD symptoms, cognition, school-related, and other.

One pharmacological and 14 non-pharmacological (54%) studies reported parent outcomes (17 different measures). Eleven melatonin studies (85%) recorded adverse events, with variation in how data were collected and reported. One non-pharmacological study reported an explicit method of collecting on adverse events. Several process measures were reported related to adherence, feasibility of delivery, acceptability and experiences of receiving the intervention.

Conclusions: There is a lack of consistency between studies in the outcome measures used to assess the effectiveness of interventions for non-respiratory sleep disturbances in children with neurodisabilities. A core outcome set, with international consensus, should be developed in consultation with parents, children and young people, and those involved in supporting families.

For peer review only - http://bmjopen.bmj.com/site/about/guidelines.xhtml

Registration number systematic review PROSPERO (CRD42016034067)

Strengths and limitations of this study

- Includes multiple interventions and populations thus providing a comprehensive overview of outcome measurement in this field.
- Provides data to inform the first stage of development of a core outcome set.
- The study provides efficient use of data from an existing review but some outcomes that were outside the scope of our effectiveness review may have been missed.

For peer review only

For peer review only - http://bmjopen.bmj.com/site/about/guidelines.xhtml

INTRODUCTION

Child sleep problems are associated with poorer educational outcomes and daytime behaviour difficulties[1] as well as having a negative impact on parents, such as heightened levels of parental stress and irritability.[2] Sleep disturbance can impact on all members of the family and it is often the parents' own poor sleep quality which leads to them seeking help with their child's sleep. [3] Sleep disturbances are more common and severe in children with neurodisabilities compared with typically developing children.[4, 5] A recent national research prioritisation exercise ranked management of sleep disturbance for this group of children in the top ten research priorities.[6]

In response to a commissioned call from the UK National Institute for Health Research (NIHR), we undertook a systematic review, to evaluate the effectiveness of pharmacological and non-pharmacological interventions for children with neurodisabilities experiencing non-respiratory sleep disturbances. The NIHR commissioning brief requested a broad systematic review to 'take stock' of the current available evidence in order to inform future research.

One of the challenges we faced while undertaking the review was the diversity of outcomes assessed across the included studies, possibly a reflection of the fact that there is no agreed set of outcomes recommended for use in trials assessing the effectiveness of interventions in this field of research. Based on searches of the COMET database [http://www.comet-initiative.org/ (accessed 21 June 2017)] there is no core outcome set in relation to children with neurodisabilities or typically developing children who experience non-respiratory sleep disturbances. A core outcome set is an agreed minimum, standardised set of outcomes that should be measured and reported in randomised controlled trials (RCTs) for a specific clinical area.[7] They are required to maximise comparability across studies in a specific field in order to facilitate quantitative and narrative synthesis, reduce selective outcome reporting in studies of effectiveness and increase the relevance of results from primary studies and systematic reviews. [7] An important first step, if there is not a core outcome set in a field, is to establish whether one is needed. [8] The breadth of our review, covering both pharmacological and non-pharmacological interventions provided an opportunity to explore whether a core outcome set is required.

The results of the systematic review of effectiveness are available separately (www.journalslibrary.nihr.ac.uk/programmes/hta/1421202/#/).[9] The aim of this paper is to summarise the outcome measures used in the RCTs and other effectiveness studies evaluating pharmacological and non-pharmacological interventions for the management of non-respiratory

For peer review only - http://bmjopen.bmj.com/site/about/guidelines.xhtml

sleep disturbances in children with neurodisabilities and make recommendations about whether a core outcome set is required.

METHODS

We used the included studies from our systematic review of treatment effectiveness, which was based on a prospectively registered review protocol (PROSPERO, registration number: CRD42016034067). A summary of the methods relevant to this paper is provided below; full details of the methods for the overall review will be available in a HTA report (www.journalslibrary.nihr.ac.uk/programmes/hta/1421202/#/).

Databases searched

We searched the following without language restrictions up to February 2017: Applied Social Science Abstracts & Indexes (ASSIA); The Cochrane Central Register of Controlled Trials (CENTRAL); Cochrane Database of Systematic Reviews (CDSR); Conference Proceedings Citation Index; Cumulative Index to Nursing & Allied Health (CINAHL); Database of Abstracts of Reviews of Effects (DARE); Embase; Health Management Information Consortium (HMIC); MEDLINE; MEDLINE In-Process; PsycINFO; Science Citation Index; Social Care Online; Social Policy & Practice; ClinicalTrials.gov; WHO International Clinical Trials Registry Platform (ICTRP); and the UK Clinical Trials Gateway. Full search strategies used are available from the corresponding author. The search results were loaded into EndNote bibliographic software (version 17.0.2.7390, Thomas Reuters, CO, USA).

Study selection

One researcher screened titles only to remove obviously irrelevant records (10% checked by a second researcher); two researchers then independently screened abstracts and ordered potentially relevant papers; the full papers were screened independently by two researchers. We included studies of children and young people (≤ 18 years old) with neurodisability (as defined by Morris *et al.* [10]), experiencing non-respiratory sleep disturbances, which evaluated pharmacological (melatonin, clonidine, and antihistamines) or non-pharmacological Interventions (e.g. behavioural, cognitive-behavioural, self-help resources, complementary therapies). A best available evidence approach was used with non-randomised controlled studies and before and after studies included where RCT evidence was not available.

Outcomes

A broad range of outcomes were of interest:

For peer review only - http://bmjopen.bmj.com/site/about/guidelines.xhtml

Primary outcomes: child sleep-related outcomes (including parent/carer and child reported outcomes (e.g. using sleep diaries and actigraphy); parent sleep-related outcomes (e.g. quality of sleep); and measures of perceived parenting confidence, efficacy, or understanding of sleep/sleep management (which can be a specific focus of parent training/behavioural interventions which seek to change how parents manage sleep disturbance).

Secondary outcomes: child-related quality of life, daytime behaviour and cognition; parent/carer outcomes such as quality of life and well-being, physical well-being, mental well-being; family functioning; and adverse events.

Data extraction and synthesis

For each study the specific outcome measures used were extracted with details of how the outcome was defined or described by the author (where reported) and, where relevant, the method of assessment e.g. child/parent reported outcome measure, sleep diaries, actigraphy, polysomnography. Data were extracted by one researcher and checked by a second. The full data extraction tables will be available at available at www.journalslibrary.nihr.ac.uk/programmes/hta/1421202/#/.

Outcomes were tabulated and grouped into core areas, then by outcome domain and specific outcome measure used. This was undertaken by a single researcher and discussed with the wider team. The data were described in a narrative synthesis grouped by the categories.

RESULTS

Overview of included studies

After removal of duplicates 15,745 records were screened and 387 full-text records assessed for eligibility. Thirty-nine studies (reported across 64 articles) were included: 13 studies of melatonin, published between 1996 and 2012 and 26 studies of parent-directed, environmental, dietary and complementary medicine interventions, published between 1991 and 2016 (Table 1). The latter group of studies are referred to as non-pharmacological for convenience. The studies were most commonly undertaken in the UK (36%) and USA (26%) followed by Australia (15%) and Canada (13%). Single studies were reported from the Netherlands, Italy, Israel and China. Study designs encompassed RCTs (both parallel-group and crossover), and, for the non-pharmacological interventions, controlled before and after studies, and one-arm before and after studies were also included.

For peer review only - http://bmjopen.bmj.com/site/about/guidelines.xhtml

The studies assessed five core outcome areas: child sleep, other child outcomes, parent-related, adverse events and process outcomes. All of the studies (n=39) reported at least one child sleep-related outcome: 19 (49%) reported other types of child outcomes such as daytime behaviour and cognition; and fourteen reported parent/carer outcomes (36%) (Table 1).

For the non-pharmacological studies, the first outcome measurement time points ranged from immediately post intervention to two months post intervention and five measured additional follow-up time points. All the melatonin studies had a single follow-up time point immediately following the completion of the intervention, which ranged from 10 days to 12 weeks post randomisation.

Child sleep outcomes

Table 2 reports the child sleep outcome measures used by at least one study. Fifty-four different measures of child related sleep outcomes were reported. There were five domains: global assessments of sleep (n=17 outcome measures); sleep initiation (n=10), sleep maintenance (n=18), sleep scheduling (n=4) and other outcomes (n=5).

In the melatonin studies, total sleep time (TST) was the most commonly reported global sleep outcome, reported by 12 of the 13 included studies (92%). Seven studies used both actigraphy and sleep diaries to measure this outcome and five used sleep diaries only. Of those using both methods, four reported the results from both and the others reported the actigraphy results only, using the diaries to inform or verify actigraphy data. The most common way to measure the sleep initiation domain was sleep onset latency (SOL, 85%); and within the sleep maintenance domain was night awakening (46%). As with TST, these outcomes were assessed using both actigraphy and sleep diary (reporting results of both or actigraphy only) or using sleep diary only. Sleep scheduling and parent-reported global measures of sleep were rarely reported in the melatonin studies (Table 2).

For the non-pharmacological studies, the sleep outcome measures used were more disparate with only one measure reported by more than half the studies. The most commonly reported global measure of sleep (and the most commonly reported overall) was the parent-reported Child Sleep Habits Questionnaire (CSHQ; 58%). TST calculated from actigraphy or sleep diary data was much less commonly used (39%) compared to melatonin studies. For the sleep initiation domain, the most commonly reported was SOL (42%); for sleep maintenance it was number of night awakenings (15%) and wake after sleep onset (15%); sleep scheduling was rarely reported. There was some overlap between pharmacological and non-pharmacological studies in the measures used, most commonly TST, sleep efficiency and SOL (Table 2).

Even where studies were measuring the same outcome, there was variation in how this was measured, though often the method used was not specified. For example, in terms of wake time,

For peer review only - http://bmjopen.bmj.com/site/about/guidelines.xhtml

one study used actigraphy and defined the outcome as the last epoch of actigraphically assessed immobility before the start of a 10 minute consecutive period of activity around the time of leaving bed;[11] two used parent-reported wake time using a sleep diary (no further definition provided);[12, 13] and one study used a momentary time sampling observation of children who were in-patients.[14] Wake after sleep onset (WASO) and night awakening both assess sleep maintenance, but different approaches to measurement were used. One study used actigraphy to measure night awakenings (definition not provided);[15] one used a sleep diary (defining night awakenings as the number of awakenings per week that parents were aware of);[16] and the other two did not provide a definition.[13, 17] All studies reporting WASO used actigraphy, two of which defined WASO as a sum of the all wake epochs during the sleep period (total time awake during the night excluding the sleep onset latency period)[18, 19] (two did not specify how it was defined[20, 21]). Where SOL was defined, there was also some variation. Definitions included time from lights out to sleep onset, the time from start of bedtime routine to sleep, the amount of time between when the child was put to be and they fell asleep, and the time between taking melatonin and falling asleep.

Other child outcomes

Nineteen studies reported child outcomes other than sleep (Table 3): 15 non-pharmacological studies (58%) and four pharmacological studies (31%). The domains assessed were child behaviour, quality of life, ADHD symptoms, cognition, school-related, and other. Twenty-nine different measures were used across the 18 studies and no single measure was used by more than three of the studies, though most by only one or two studies (Table 3). The measures used ranged from validated tools to study-specific questionnaires.

It was not always clear whether studies had used the same version of a measure. For example, there are two versions of the Daily Parent Rating of Evening and Morning Behaviour (DREMB) scale; [22] the original version (DREMB) and a revised version (DREMB-R) with an item on irritability removed. Both studies using this outcome measure referenced the same source for the scale suggesting they used the same version. However, this could not be confirmed as only one gave details of the score range of the version used.[20, 23]

Finally, we encountered a lack of clarity in reporting with respect to whether sub-scale and/or total scores were used. Thus, for the two studies which used the Child Behaviour Checklist, one reported the eight subscales scores plus a total score and daytime internalising behaviour problem score (calculated from scores on three of the subscales), [24] the other study presented insufficient information about the scores used. [19]

For peer review only - http://bmjopen.bmj.com/site/about/guidelines.xhtml

Adverse outcomes

Eleven melatonin studies (85%) recorded adverse events, with variation across studies in how data were collected and reported. Different approaches were described to measuring adverse events with varying levels of standardisation, including: a standardised assessment tool which classified events into seven domains;[25] elicitation of adverse events during in-person/phone-call visits by the study team;[12, 26] using study specific questionnaires completed by parents;[27] [28] one reported using a standardised form;[29] open-ended interviews;[11, 30] one reported by parents using an unspecified method;[31] and in one study it was unclear how the data were collected.[32] One non-pharmacological study reported a specific method of collecting on adverse events: making a 24 hour telephone line available to parents for reporting adverse events and gathering the information at weekly parents reviews (face-to-face or telephone). [15] While other non-pharmacological studies reported some adverse events it was unclear whether there was a systematic method of gathering this information.

Parent outcomes

Fourteen non-pharmacological studies (54%) reported parent outcomes (Table 4). A range of outcome domains were assessed, and measures used were not consistent. Domains included parental mental health (three outcome measures across 5 studies), parenting (for example, perceived confidence, efficacy or knowledge in relation to managing their child's sleep problem) (five outcome measures across 9 studies), quality of parental sleep (six outcome measures across 3 studies) and a range of other outcomes including attendance at work and locus of control (three measures across four studies). One melatonin study assessed parent outcomes; specifically, it used the Epworth Sleepiness Scale to assess parental daytime sleepiness.[25]

Process outcomes

Several other outcomes and process measures were reported related to adherence, feasibility of delivery, acceptability and experiences of receiving the intervention. These included: adherence to treatment (based on medication count (pharmacological studies only), intervention sessions attended, adherence to specific components of the intervention; withdrawal from study); various study-specific individual items or questionnaires to ascertain parents views about specific aspects of the intervention or overall views, for example, the proportion of participants rating treatment on a five point rating scale from "too tough" to "too soft"; rating of overall treatment helpfulness (visual analogue scale) and specific components of the intervention; study specific Caregivers Acceptance of Treatment Survey (Likert Scale response format); a modified version of the Program Evaluation

For peer review only - http://bmjopen.bmj.com/site/about/guidelines.xhtml

Questionnaires and qualitative interviews. There was variation in the extent to which this information was reported in a standardised way, for example limited detail in the methods used.

For peer review only

For peer review only - http://bmjopen.bmj.com/site/about/guidelines.xhtml

Table 1 Summary of included studies

Study	Year	Country	Intervention	Study design	Child outcomes		Parent outcomes	Adverse events
					Sleep	Other		
			**MELATONIN**					
**Appleton *et al.* [25]**	2012	UK	Melatonin	Parallel RCT	X	X	X	X
**Camfield *et al.*[33]**	1996	Canada	Melatonin	N of 1 crossover RCT	X			
**Cortesi *et al.*[26]**	2012	Italy	Melatonin	Parallel RCT	X	X		X
**der Heijden *et al.*[11]**	2007	Netherlands	Melatonin	Parallel RCT	X	X		X
**Dodge and Wilson [28]**	2001	USA	Melatonin	Crossover RCT	X			X
**Garstang and Wallis [34]**	2006	UK	Melatonin	Crossover RCT	X			
**Hancock *et al.* [31]**	2005	UK	Melatonin	Crossover RCT	X			X
**Jain *et al.*[12]**	2015	USA	Melatonin	Crossover RCT	X	X		X
**Jan *et al.*[35]**	2000	Canada	Melatonin	Crossover RCT	X			X
**Wasdell *et al.* [30]**	2008	Canada	Melatonin	Crossover RCT	X			X
**Weiss *et al.*[29]**	2006	Canada	Melatonin	Crossover RCT	x	X		X
**Wirojanan *et al.*[32]**	2009	USA	Melatonin	Crossover RCT	X			X
**Wright *et al.*[27]**	2011	UK	Melatonin	Crossover RCT	X			X
			**NON-PHARMACOLOGICAL**					
**Adkins *et al.*[18]**	2012	USA	Parent-directed, non-tailored	Parallel RCT	X			
**Austin *et al.*[13]**	2013	Australia	Parent-directed, tailored	Before-after	X	X		
**Beresford *et al.*[3]**	2012	UK	Parent-directed, tailored	Parallel RCT	X		X	
**Beresford, *et al.*[3], Intervention 2**	2012	UK	Parent-directed, tailored	Before-after	X		X	
**Beresford *et al.*[3] Intervention 3**	2012	UK	Parent-directed non-tailored	Before-after	X		X	
**Beresford *et al.*[3] Intervention 4**	2012	UK	Parent-directed non-tailored	Before-after	X		X	
**Bramble, (1997) [36]**	1997	UK	Parent-directed non-tailored	Before-after	X	X	X	
**Francis and Dempster[37]**	2002	Australia	Valerian	Crossover RCT	X	X		

For peer review only

For peer review only - http://bmjopen.bmj.com/site/about/guidelines.xhtml

Study	Year	Country	Intervention	Study design	Child outcomes		Parent outcomes	Adverse events
					Sleep	Other		
**Gringras, *et al.*[38]**	2014	UK	Weighted blanket	Crossover RCT	X	X		X
**Guilleminault, *et al.*[39]**	1993	USA	Light therapy+behavioural programme	Before-after	X			
**Hiscock *et al.*[20]**	2015	Australia	Parent-directed, tailored	Parallel RCT	X	X	X	
**Johnson, *et al.*[40]**	2013	USA	Parent-directed, tailored	Parallel RCT	X			
**Malow *et al.*[19]**	2014	USA	Parent-directed non-tailored	Parallel RCT	X	X		
**Montgomery et al.[41]**	2004	UK	Parent-directed non-tailored	Parallel RCT	X			
**Moss *et al.*[42]**	2014	Australia	Parent-directed, tailored	Parallel RCT	X	X	X	
**Oriel *et al.*[17]**	2016	USA	Aquatic exercise programme	A-B-A	X			
**Peppers *et al.*[43]**	2016	USA	Non-comprehensive, parent-directed	Before-after	X	X	X	
**Piazza *et al.*[44]**	1997	USA	Behavioural	Parallel RCT	X			
**Quine and Wade[45]**	1991	UK	Parent-directed, tailored	Before-after	X	X	X	
**Reed *et al.*[21],**	2009	Canada	Parent-directed non-tailored	Before-after	X	X	X	
**Sciberras *et al.*[23]**	2011	Australia	Parent-directed, tailored	Parallel RCT	X	X	X	
**Weiskop, *et al.*[16]**	2005	Australia	Parent-directed, tailored	Before-after	X			
**Wiggs and Stores[46]**	1998	UK	Non-comprehensive, parent-directed	RCT	X	X	X	
**Yehuda *et al.*[47]**	2011	Israel	Essential fatty acids	Controlled before-after	X	X		X
**Yu *et al.*[24]**	2015	UK	Parent-directed non-tailored	Before-after	X	X	X	
**Yu and Hong[48]**	2012	China	Acupuncture and earpoint taping	Before-after	X			

[a] definitions for how studies were classified as parent directed and tailored/non-tailored are available in the effectiveness review report (www.journalslibrary.nihr.ac.uk/programmes/hta/1421202/#/)

For peer review only - http://bmjopen.bmj.com/site/about/guidelines.xhtml

Table 2 Child Sleep related outcomes

		Melatonin(n=13)		Non-pharmacological (n=26)		All studies (n=39)
	**Sleep outcome**	**Number of studies (%)**	**Studies measuring outcome**	**Number of studies (%)**	**Studies measuring outcome**	**Number of studies (%)**
Global measures	Total sleep time	12 (92)	[11, 12, 25-34]	11 (42)	[15-21, 37, 39, 40, 46]	22 (59)
	Sleep efficiency	5 (38)	[11, 12, 25, 26, 30]	5 (19)	[15, 18-20, 40]	10 (26)
	Longest sleep episode	1 (8)	[30]	0 (0)		1 (3)
	Changes in sleep pattern	1 (8)	[35]	0 (0)		1 (3)
	CSHQ	1 (8)	[26]	15 (58)	[3, 13][a, b, c,d] [15, 17, 19-21, 23, 24, 42, 43, 48]	16 (41)
	SBQ	1 (8)	[12]	0 (0)		1 (3)
	Parent-set Child Sleep Goals	0 (0)		4 (15)	[3] [a, b, c, d]	4 (10)
	Composite Sleep Index (from modified version of Simonds and Parraga Sleep Questionnaire)	0 (0)		3 (12)	[15, 40, 46]	3 (8)
	Goal Attainment Scale	0 (0)		2 (8)	[16, 42]	2 (5)
	Change in goal attainment rating	0 (0)		1 (4)	[3]	1 (3)
	Sleep problems over past 4 weeks	0 (0)		1 (4)	[20]	1 (3)
	Family Inventory of Sleep Habits	0 (0)		3 (12)	[19, 21, 24]	3 (8)
	Composite Sleep Disturbance Score	0 (0)		1 (4)	[41]	1 (3)
	Sleep quality (visual analogue scale)	0 (0)		1 (4)	[37]	1 (3)
	Sleep improvement – child and parent rating scale	0 (0)		1 (4)	[15]	1 (3)

For peer review only - http://bmjopen.bmj.com/site/about/guidelines.xhtml

	Sleep outcome	Melatonin(n=13) Number of studies (%)	Melatonin(n=13) Studies measuring outcome	Non-pharmacological (n=26) Number of studies (%)	Non-pharmacological (n=26) Studies measuring outcome	All studies (n=39) Number of studies (%)
	Quality of sleep – child smiley face rating	0 (0)		1 (4)	[15]	1 (3)
	Quality of sleep – Likert rating scale	0 (0)		1 (4)	[47]	1 (3)
Sleep initiation	Sleep onset latency	11 (85)	[11, 12, 25-32, 34]	10 (39)	[13, 15-19, 21, 36, 37, 40]	21 (54)
	Bedtime	2 (15)	[12, 26]	1 (4)	[21]	3 (8)
	Sleep onset	2 (15)	[11, 32]	1 (4)	[14]	3 (8)
	Bedtime resistance	0 (0)		1 (4)	[13]	1 (3)
	Bedtime routine	0 (0)		1 (4)	[13]	1 (3)
	Number of pre-sleep disturbances	0 (0)		1 (4)	[16]	1 (3)
	Falling asleep in own bed	0 (0)		1 (4)	[16]	1 (3)
	Difficulty falling asleep (single item Likert scale)	1 (8)	[11]	0 (0)		1 (3)
	Severity of bedtime settling problems (Behaviour Screening Questionnaire)			1(4)	[45]	1 (3)
	Time to settle at night	0 (0)		2 (7.7)	[16, 45]	2 (5)
Sleep maintenance	Night awakenings	6 (46)	[27, 28, 30-33]	5 (15)	[13, 15-17, 44]	11 (28)
	Number of nights without waking	1 (8)	[33]	0 (0)		1 (3)
	Wake time	2 (15)	[11, 12]	2 (8)	[13, 14]	4 (10)
	Time spent moving during sleep period	1 (15)	[11]	0 (0)		1 (3)
	Co-sleeping	0 (0)		2 (8)	[13, 16]	2 (5)
	Number of nights not sleeping in own bed			1 (4)	[45]	1 (3)

For peer review only - http://bmjopen.bmj.com/site/about/guidelines.xhtml

	Sleep outcome	Melatonin(n=13) Number of studies (%)	Melatonin(n=13) Studies measuring outcome	Non-pharmacological (n=26) Number of studies (%)	Non-pharmacological (n=26) Studies measuring outcome	All studies (n=39) Number of studies (%)
	Wake After Sleep Onset	2 (15)	[12, 26]	4 (15)	[18-21]	6 (15)
	Severity of sleep problem (VAS) (incorporating disturbed sleep)	0 (0)		1 (4)	[36]	1 (3)
	Movement during sleep	0 (0)		1 (4)	[46]	1 (3)
	Movement index	0 (0)		1 (4)	[46]	1 (3)
	Fragmentation index	0 (0)		1 (4)	[46]	1 (3)
	Time spent awake during night	0 (0)		3 (12)	[15, 37, 45]	3 (8)
	Proportion of nights with > 1 wakening	0 (0)		1 (4)	[15]	1 (3)
	Parent/caregiver reported 'no' or 'mild' sleep problems	0 (0%)		1 (3.8%)	[23]	1 (2.6%)
	Longest wake and sleep periods during 24 hour cycle	0 (0)		1 (4)	[39]	1 (3)
	Time of night wakes and return to sleep	0 (0)		1 (4)	[14]	1 (3)
	Hours of disturbed sleep	0 (0)		1 (4)	[14]	1 (3)
	Sleep Disturbance Index	0 (0)		1 (4)	[13]	1 (3)
Sleep scheduling	Naptime	1 (8)	[26]	0 (0)		1 (3)
	Napping	0 (0)		1 (4)	[13]	1 (3)
	Distribution of sleep bouts during 24 hour cycle	0 (0)		1 (4)	[39]	1 (3)
	Degree of fatigue during the day – Likert rating scale	0 (0)		1 (4)	[47]	1 (3)
	Interdaily stability	1 (8)	[11]	0 (0)		1 (3)
Other	Interdaily variability	1 (8)	[11]	0 (0)		1 (3)
	L5	1 (8)	[11]	0 (0)		1 (3)

For peer review only - http://bmjopen.bmj.com/site/about/guidelines.xhtml

	Melatonin(n=13)		Non-pharmacological (n=26)		All studies (n=39)
**Sleep outcome**	**Number of studies (%)**	**Studies measuring outcome**	**Number of studies (%)**	**Studies measuring outcome**	**Number of studies (%)**
Arousal Index (AASM)	1 (8)	[12]	0 (0)		1 (3)
Percentage of time in each sleep stage	1 (8)	[12]	0 (0)		1 (3)

AASM Arousal Index; CSHQ Children's Sleep Habits Questionnaire; SBQ Sleep Behaviour Questionnaire; VAS visual analogue scale [a] Beresford, Intervention 1, [b] Beresford Intervention 2, [c] Beresford Intervention 3

Table 3 Other child outcomes

		Melatonin (n=13)		Non-pharmacological (n=26)		All (n=39)
	**Outcome**	**Number of studies (%)**	**Studies measuring outcome**	**Number of studies (%)**	**Studies measuring outcome**	**Number of studies (%)**
Behaviour	Daily Parent Rating of Evening and Morning Behaviour Scale	0 (0)		2 (8)	[20, 23]	2 (5)
	Aberrant Behaviour Checklist	1 (8)	[25]	2 (8)	[15, 46]	3 (8)
	Behaviour Problem Index	0 (0)		2 (8)	[36, 45]	2 (5)
	Child Behaviour Checklist	1 (8)	[11]	2 (8)	[19, 24]	3 (8)
	Repetitive Behaviour Scale – Revised	0 (0)0		2 (8)	[19, 21]	2 (5)
	Child daytime behaviour (based on diary)	0 (0)		1 (4)	[37]	1 (3)
	Developmental Behaviour Checklist-Parent Version	0 (0)		2 (8)	[13, 42]	2 (5)
	Sensory Behaviour Questionnaire	0 (0)		1 (4)	[15]	1 (3)

For peer review only - http://bmjopen.bmj.com/site/about/guidelines.xhtml

	Outcome	Melatonin (n=13) Number of studies (%)	Studies measuring outcome	Non-pharmacological (n=26) Number of studies (%)	Studies measuring outcome	All (n=39) Number of studies (%)
	(unpublished)					
	Behaviour Assessment System for Children-Parent Rating Scale	1 (8)	[12]	0 (0)		1 (3)
Quality of life	Paediatric Quality of Life Inventory 4.0	1 (8)	[25]	3 (12)	[19, 20, 23]	4 (10)
	Quality of Life in Childhood Epilepsy	1 (8)	[12]	0 (0)		1 (3)
	Level of good mood in general (Likert scale)	1 (8)	[29]	1 (4)	[47]	2 (5)
	TNO-AZL Quality of Life Questionnaire	1 (8)	[11]	0 (0)		1 (3)
ADHD symptoms	Vanderblit ADHD Symptom Checklist	0 (0)		1 (4)	[43]	1 (3)
	ADHD Rating Scale IV	0 (0)		2 (8)	[20, 23]	2 (5)
	Conner's Attention Deficit Scale-Parent Version	1 (8)	[29]	0 (0)		1 (3)
Cognitive	Ability to concentrate during the day, mainly at school (Likert scale)	0 (0)		1 (4)	[47]	1 (3)
	Working memory test battery[a]	0 (0)		1 (4)	[20]	1 (3)
	Sustained attention dots task-completion time	1 (8)	[11]	0 (0)		1 (3)
	Sustained attention dots task-inaccuracy[b]	1 (8)	[11]	0 (0)		1 (3)
	Erikson Task – reaction time and error incidence[c]	1 (8)	[11]	0 (0)		1 (3)
School-related	Homework completion (Likert scale)	0 (0)		1 (4)	[47]	1 (3)
	School attendance	0 (0)		2 (8)	[20, 23]	2 (5)

For peer review only - http://bmjopen.bmj.com/site/about/guidelines.xhtml

	Outcome	Melatonin (n=13) Number of studies (%)	Studies measuring outcome	Non-pharmacological (n=26) Number of studies (%)	Studies measuring outcome	All (n=39) Number of studies (%)
	Teacher's Report Form	1 (8)	[11]	0 (0)		1 (3)
Other	Meal times	0 (0)		1 (4)	[13]	1 (3)
	Parent report of other professional help sought	0 (0)		1 (4)	[20]	1 (3)
	Parental Concerns Questionnaire	0 (0)		1 (4)	[21, 24]	1 (3)
	Strength and Difficulties Questionnaire	0 (0)		1 (4%)	[20]	1 (3)
	Parent and teacher reported core problems[d]	1 (8)	[11]	0 (0)		1 (3)

[a]Backwards digit recall, counting recall, listening recall; [b]Percentage of misses plus false alarms relative to the total number of trials; [c]Reaction time and error incidence between congruent and incongruent tasks; [d]Core problems were spontaneously and individual defined by parents

For peer review only - http://bmjopen.bmj.com/site/about/guidelines.xhtml

Table 4 Parent/carer outcomes (non-pharmacological studies n=26)

Domain	Outcome Measure	Number of studies (%)	Studies measuring outcome
Parental mental health and well-being	Depression Anxiety Stress Scales	2 (8)	[20, 23]
	The Parenting Stress Index-Short Form	3 (12)	[21, 24, 42]
	The Malaise Inventory	3 (12)	[36, 45, 46]
Parenting (e.g. Perceived confidence efficacy or knowledge)	Perceived ability to control own and partner's sleep difficulties (visual analogue scale)	1 (4)	[46]
	Parental satisfaction with their ability to cope with their child's sleep (Likert scale)	1 (4)	[46]
	Parent satisfaction with child's sleep	1 (4)	[46]
	Parenting Sense of Competence scale (plus satisfaction and efficacy subscales)	5 (19)	[3][a,b,c,d] [19]
	Knowledge of Behavioural Principles as Applied to Children	1 (4)	[45]
Parental sleep	Maternal Sleep Scale	1 (4)	[36]
	Pittsburgh Sleep Quality Index	1 (4)	[24]
	Maternal total sleep time (actigraphy)	1 (4)	[46]
	Other actigraphy measures of sleep (sleep period, activity score, movement index, fragmentation index)	1 (4)	[46]
	Epworth Sleepiness Scale	1 (4)	[46]
	Parental satisfaction with own sleep (Likert scale)	1 (4)	[46]
Other	Work attendance (number of days missed or late for work)	2 (8)	[20, 23]
	Parent Satisfaction Likert Survey	1 (4)	[43]
	Parental locus of Control	1 (4)	[46]

For peer review only - http://bmjopen.bmj.com/site/about/guidelines.xhtml

DISCUSSION

Principal findings

The findings suggest a strong imperative to develop a core outcome set to be used in trials in this field. Over 70 different outcome measures were reported across 39 studies, the vast majority of measures reported by three or fewer studies. This greatly impedes analysis of data in systematic reviews due to lack of comparability across studies. It also makes it difficult to assess the likelihood of selective outcome reporting, with the associated risks of overestimating intervention effectiveness. It is unlikely that all these measures have equal importance for children and parents, leading to uncertainty when making judgements about the relevance of specific study results.

There were five core areas assessed in the included studies: child sleep (six domains); other child outcomes (five domains); parent outcomes (four domains) adverse events and process measures. This suggests some consensus at the broadest level about what is relevant to measure; however, as the classification became more granular the diversity increased.

There was overlap between pharmacological and non-pharmacological studies in terms of the child sleep domains assessed and, to some extent, the measures used, reflecting the fact that, regardless of mechanism, a key target of both types of intervention was some aspect of child sleep. However, compared to the melatonin studies, there was more focus in the non-pharmacological studies on the core areas of child non-sleep, parent outcomes and family experience. This reflects the complex nature of these interventions, with parent understanding and perceived competency in managing their child's sleep being implicit in the mechanism of action. The parent outcome domains were mental health, parenting (perceived confidence, efficacy, or knowledge) and quality of sleep. Arguably, it is surprising that more studies of parent-directed interventions did not assess impact on parental perceived competence/confidence and that melatonin studies have not taken as family-centred approach to assessing the effects of the intervention in this population.

The most widely used outcome measure in the melatonin studies was total sleep time (measured using actigraphy and/or sleep diary) and in the non-pharmacological studies was the CSHQ. Although they are both within the core domain of child sleep and provide a global assessment, they differ considerably in level of objectivity, aspects of sleep assessed, feasibility and cost.[49] They could be viewed as complementary measures to be used together, though only six studies used both. Even amongst these more common outcome measures there was variation between studies in how they were used. Lack of standard scoring rules and variation in definitions used for actigraphy-derived outcomes has been identified as an area of concern, which is supported by our research. [50]

For peer review only - http://bmjopen.bmj.com/site/about/guidelines.xhtml

Adverse events were reported in the majority of melatonin trials but appeared to be rarely considered in the non-pharmacological studies. Standardisation of the reporting and data collection methods for adverse events in future trials is important to understand the safety of pharmacological interventions. This also has relevance for non-pharmacological studies as well, where interventions may have unintended consequences. [51-53]

The diversity of outcome measures used significantly limited both the narrative and quantitative syntheses conducted for the review of the effectiveness of these interventions.[9] Our findings are consistent with the results of studies in other fields exploring the extent of consistency of outcome measures used and in other evidence syntheses.[54, 55]

Unanswered questions and future research

A core outcome set would greatly assist the usefulness of research in this field, allowing comparison between studies and also ensuring that the outcomes that are assessed are of relevance to children, their parents and also the healthcare professionals and others involved in providing support. Although sleep has been identified as an important outcome by young people with neurodisabilities and their parents it remains unclear what aspects of sleep are most important to them, for example whether it is the child's total amount of sleep time, when they sleep, how many times they wake during the night etc. The importance to them of improving other outcomes such as child daytime behaviour or parental outcomes as part of sleep management interventions and which should be prioritised is also unclear.[56]

Effectiveness research that does not assess relevant and important outcomes is a source of avoidable waste.[57] The core outcome set should be developed in consultation with parents and carers, children themselves where possible, and healthcare professionals and others involved in supporting parents and children, using a structured process such as that developed by the COMET Group.[8] It is currently unclear whether the outcomes being assessed in studies evaluating sleep disturbance interventions are those that are important to families. Research in other fields has shown a disparity between the outcomes important to patients and those that are assessed in intervention studies. [55, 58, 59] Development of a core outcome set should be international in scope to ensure future widespread adoption. In addition to identifying core outcomes, consensus will be required regarding appropriate measurement instruments. This should draw on appropriate methods to ensure selection of reliable and valid measures such as those used by the COSMIN initiative.[60] Factors such as feasibility and respondent burden will also be important to consider. The heterogeneity in outcome measures used by studies in our review was compounded by the

For peer review only - http://bmjopen.bmj.com/site/about/guidelines.xhtml

variation across studies in the clarity of information provided on how some outcomes were measured. Future research should follow appropriate reporting guidelines.[61]

A key decision point at the outset would be whether a core outcome set should be developed separately for pharmacological and non-pharmacological interventions. We suggest that an appropriate approach would be to have a core outcome set common across interventions with additional outcomes agreed where relevant for the two types of interventions. For parent-directed interventions, children's sleep outcomes are mediated by parent outcomes achieved by the intervention (for example, parental acquisition of new knowledge and understanding of sleep, training in managing sleep disturbance). Formalising and refining a theory of change for such interventions would support and inform the development of a core outcome set. This is relevant for both child and parent outcomes.

Consistency in follow-up time points across studies of similar interventions, at least at the primary follow-up point, is required in order to support comparisons between studies in future systematic reviews and meta-analyses. This will require reaching consensus with regard to the most clinically meaningful follow-up time points, and whether evaluations should seek to also investigate maintenance of outcomes. Again, for non-pharmacological studies in particular, this would benefit from being based on an evidence-informed theory of change as the implementation of newly acquired knowledge and skills in managing a child's sleep may take time to have an effect. The final follow-up time-point for most studies in our effectiveness review only allowed consideration of short term outcomes. Future studies need to consider what longer-term follow-ups should be incorporated into study designs.

Strengths and weaknesses of the study

The systematic review included a wide range of neurodisabilities and interventions in children aged 18 years or under. It therefore provides a comprehensive overview of the outcome domains being assessed in this field of research and the outcome measures being used. However, we may have under-estimated the range of outcomes being assessed as this work was undertaken as part of a systematic review of effectiveness. Thus, although the outcomes of interest were very broad we did not exhaustively include all outcomes; therefore, we may have failed to identify some outcomes, for example some physiological aspects of sleep. In addition, we assigned the measures to one of a number of outcome domains, others doing the same task may allocate differently or attribute to different domains. However, these factors are unlikely to change the conclusions. The description of outcome domains and measures used provides important evidence for the field in moving towards developing a core outcome set. While these will contribute to the development of a list of outcomes

For peer review only - http://bmjopen.bmj.com/site/about/guidelines.xhtml

for a Delphi survey, as a first stage of developing a core outcome set, it is important that this is supplemented by the views of parents and children, healthcare professionals involved in designing and delivering interventions and researchers working in the field.

CONCLUSION

There is a lack of consistency between studies in the outcome measures used to assess the effectiveness of interventions for non-respiratory sleep disturbances in children with neurodisabilities. This hampers evidence synthesis and creates uncertainty about the relevance of study findings to parents and children. A core outcome set, with international consensus, should be developed in consultation with parents, children and young people, and those involved in supporting families.

FUNDING STATEMENT

This work is derived from a project funded by the National Institute for Health Research (NIHR) HTA Programme (project number 14/212/02) and will be published in full in Health Technol Assess. Further information available at https://www.journalslibrary.nihr.ac.uk/programmes/hta/1421202/#/

This report presents independent research commissioned by the NIHR. The views and opinions expressed by authors in this publication are those of the authors and do not necessarily reflect those of the NHS, the NIHR, MRC, CCF, NETSCC, the HTA Programme or the Department of Health

COMPETING INTERESTS

Bryony Beresford and Megan Thomas were authors of studies included in the review, but were not involved in data extraction or quality assessment of these studies. Catriona McDaid is a member of the NIHR HTA & EME Editorial Board. Catherine Hewitt is a member of HTA Commissioning Board.

For peer review only - http://bmjopen.bmj.com/site/about/guidelines.xhtml

AUTHORS CONTRIBUTIONS

Catriona McDaid: jointly responsible for writing the protocol and had shared responsibility for co-ordinating and leading the project; provided advice and input to all elements of the project. Wrote sections of main report and wrote and revised drafts of this paper

Adwoa Parker and Arabella Scantlebury: equally contributed to study selection, data extraction, quality assessment and synthesis and report writing of the main report. Commented on drafts of this paper.

Caroline Fairhurst: conducted the data analysis, contributed to the report writing and commented on drafts of this paper.

Catherine Hewitt: contributed to the protocol, provided methodological advice throughout the project, commented on drafts of the main report and drafts of this paper.

Gemma Spiers: worked on the project February – September 2016. During that time she was responsible for the day-to-day running of the project and led on screening, retrieval, data extraction and quality appraisal. Commented on drafts of this paper.

Vicki Dawson, Heather Elphick, Megan Thomas: provided expert clinical advice throughout the project, contributed to screening and data extraction processes and to drafts of the report, commented on drafts of this paper.

Bryony Beresford: jointly responsible for writing the review protocol and had shared responsibility for co-ordinating and leading the project; provided advice and input to all elements of the project. Wrote sections of main report and commented on drafts of this paper.

DATA SHARING STATEMENT

The data are derived from published studies and are publicly available in the main body and appendices of the HTA Monograph.

For peer review only

REFERENCES

1. Simola, P., *Sleep problems and their implications from preschool to school age*. Studies in Educational SCiences. 2014: Käyttäytymistieteiden laitos (Helsingin yliopisto).
2. Doo, S. and Y.K. Wing, *Sleep problems of children with pervasive developmental disorders: correlation with parental stress.* Developmental medicine and child neurology, 2006. **48**(8): p. 650-655.
3. Beresford, B., et al., *Interventions E: Individually Delivered Sleep Management Interventions*, in *Managing behaviour and sleep problems in disabled children: An investigation into the effectiveness and costs of parent-training interventions*. 2012, University of Kent: London.
4. Tietze, A.L., et al., *Sleep disturbances in children with multiple disabilities.* Sleep Med Rev, 2012. **16**(2): p. 117-27.
5. Dorris, L., et al., *Sleep problems in children with neurological disorders.* Dev Neurorehabil, 2008. **11**(2): p. 95-114.
6. Morris, C., et al., *Setting research priorities to improve the health of children and young people with neurodisability: a British Academy of Childhood Disability-James Lind Alliance Research Priority Setting Partnership.* BMJ open, 2015. **5**(1): p. e006233.
7. Williamson, P.R., et al., *Developing core outcome sets for clinical trials: issues to consider.* Trials, 2012. **13**(132).
8. Williamson, P.R., et al., *The COMET Handbook: version 1.0.* Trials, 2017. **18**(3): p. 280.
9. Scantlebury, A., et al., *Non-pharmacological interventions for non-respiratory sleep disturbance in children with neurodisabilities: a systematic review.* Developmental Medicine & Child Neurology, 2018.
10. Morris, C., et al., *Towards a definition of neurodisability: a Delphi survey.* Dev Med Child Neurol, 2013. **55**(12): p. 1103-8.
11. der Heijden, K.B.V.A.N., et al., *Effect of melatonin on sleep, behavior, and cognition in ADHD and chronic sleep-onset insomnia.* J. Am. Acad. Child Adolesc. Psychiatry, 2007. **46**(2): p. 233-241.
12. Jain, S.V., et al., *Melatonin improves sleep in children with epilepsy: a randomized, double-blind, crossover study.* Sleep Med., 2015. **16**(5): p. 637-644.
13. Austin, K., J.E. Gordon, and A. O'Connell, *Preliminary Evaluation of Sleepwise Program for Children With Sleep Disturbance and Developmental Delay.* Child Fam. Behav. Ther., 2013. **35**(3): p. 195-211.
14. Piazza, C.C. and W. Fisher, *A faded bedtime with response cost protocol for treatment of multiple sleep problems in children.* J. Appl. Behav. Anal., 1991. **24**(1): p. 129-140.
15. Gringras, P., et al., *Weighted blankets and sleep in autistic children - a randomised controlled trial.* J. Sleep Res., 2014. **23**(Suppl.): p. 320-320.
16. Weiskop, S., A. Richdale, and J. Matthews, *Behavioural treatment to reduce sleep problems in children with autism or fragile X syndrome.* Dev. Med. Child Neurol., 2005. **47**(2): p. 94-104.
17. Oriel, K.N., et al., *The Impact of Aquatic Exercise on Sleep Behaviors in Children With Autism Spectrum Disorder.* Focus on Autism & Other Developmental Disabilities, 2016. **31**(4): p. 254-261.
18. Adkins, K.W., et al., *Effects of a standardized pamphlet on insomnia in children with autism spectrum disorders.* Pediatrics, 2012. **130 Suppl 2**: p. S139-44.
19. Malow, B.A., et al., *Parent-based sleep education for children with autism spectrum disorders.* J. Autism Dev. Disord., 2014. **44**(1): p. 216-228.
20. Hiscock, H., et al., *Impact of a behavioral sleep intervention on ADHD symptoms, child sleep and parent health: a randomized controlled trial.* BMJ, 2015. **350**: p. h68.
21. Reed, H.E., et al., *Parent-based sleep education workshops in autism.* J. Child Neurol., 2009. **24**(8): p. 936-945.

For peer review only - http://bmjopen.bmj.com/site/about/guidelines.xhtml

22. Faraone, S.V., A. Childress, and S.B. Wigal, *Reliability and validity of the Daily Parent Rating of Evening and Morning Behaviour Scale, Revised.* Journal of Attention Disorders, 2015.
23. Sciberras, E., et al., *Managing sleep problems in school aged children with ADHD: a pilot randomised controlled trial.* Sleep Med., 2011. **12**(9): p. 932-935.
24. Yu, X.T., et al., *Extended Parent-based Behavioural Education Improves Sleep in Children with Autism Spectrum Disorder.* HK J Paediatr (New Series), 2015. **20**(4): p. 219-225.
25. Appleton, R.E., et al., *The use of MElatonin in children with neurodevelopmental disorders and impaired sleep: a randomised, double-blind, placebo-controlled, parallel study (MENDS).* Health Technol. Assess., 2012. **16**(40): p. i-239.
26. Cortesi, F., et al., *Controlled-release melatonin, singly and combined with cognitive behavioural therapy, for persistent insomnia in children with autism spectrum disorders: a randomized placebo-controlled trial.* J. Sleep Res., 2012. **21**(6): p. 700-709.
27. Wright, B., et al., *Melatonin versus placebo in children with autism spectrum conditions and severe sleep problems not amenable to behaviour management strategies: a randomised controlled crossover trial.* J. Autism Dev. Disord., 2011. **41**(2): p. 175-184.
28. Dodge, N.N. and G.A. Wilson, *Melatonin for treatment of sleep disorders in children with developmental disabilities.* J. Child Neurol., 2001. **16**(8): p. 581-584.
29. Weiss, M.D., et al., *Sleep hygiene and melatonin treatment for children and adolescents with ADHD and initial insomnia.* J. Am. Acad. Child Adolesc. Psychiatry, 2006. **45**(5): p. 512-519.
30. Wasdell, M.B., et al., *A randomized, placebo-controlled trial of controlled release melatonin treatment of delayed sleep phase syndrome and impaired sleep maintenance in children with neurodevelopmental disabilities.* J. Pineal Res., 2008. **44**(1): p. 57-64.
31. Hancock, E., F. O'Callaghan, and J.P. Osborne, *Effect of melatonin dosage on sleep disorder in tuberous sclerosis complex.* J. Child Neurol., 2005. **20**(1): p. 78-80.
32. Wirojanan, J., et al., *The efficacy of melatonin for sleep problems in children with autism, fragile X syndrome, or autism and fragile X syndrome.[Erratum appears in J Clin Sleep Med. 2010 Aug 15;6(4):preceding 311].* J. Clin. Sleep Med., 2009. **5**(2): p. 145-150.
33. Camfield, P., et al., *Melatonin appears ineffective in children with intellectual deficits and fragmented sleep: six" N of 1" trials.* J. Child Neurol., 1996. **11**(4): p. 341-343.
34. Garstang, J. and M. Wallis, *Randomized controlled trial of melatonin for children with autistic spectrum disorders and sleep problems.* Child Care Health Dev., 2006. **32**(5): p. 585-589.
35. Jan, J.E., et al., *Clinical trials of controlled-release melatonin in children with sleep--wake cycle disorders.* J. Pineal Res., 2000. **29**(1): p. 34-39.
36. Bramble, D., *Rapid-acting treatment for a common sleep problem.* Developmental Medicine & Child Neurology, 1997. **39**(8): p. 543-547.
37. Francis, A.J.P. and R.J.W. Dempster, *Effect of valerian, Valeriana edulis, on sleep difficulties in children with intellectual deficits: randomised trial.* Phytomedicine, 2002. **9**(4): p. 273-279.
38. Gringras, P., et al., *Weighted Blankets and Sleep in Autistic Children—A Randomized Controlled Trial.* Pediatrics, 2014. **134**(2): p. 298-306.
39. Guilleminault, C., et al., *Light therapy as treatment of dyschronosis in brain impaired children.* Eur. J. Pediatr., 1993. **152**(9): p. 754-759.
40. Johnson, C.R., et al., *Behavioral parent training to address sleep disturbances in young children with autism spectrum disorder: a pilot trial.* Sleep Med., 2013. **14**(10): p. 995-1004.
41. Montgomery, P., G. Stores, and L. Wiggs, *The relative efficacy of two brief treatments for sleep problems in young learning disabled (mentally retarded) children: a randomised controlled trial.* Arch. Dis. Child., 2004. **89**(2): p. 125-130.
42. Moss, A.H.B., J.E. Gordon, and A. O'Connell, *Impact of sleepwise: an intervention for youth with developmental disabilities and sleep disturbance.* J. Autism Dev. Disord., 2014. **44**(7): p. 1695-1707.
43. Peppers, K.H., et al., *An Intervention to Promote Sleep and Reduce ADHD Symptoms.* Journal of Pediatric Health Care, 2016. **30**(6): p. e43-e48.

For peer review only - http://bmjopen.bmj.com/site/about/guidelines.xhtml

44. Piazza, C.C., W.W. Fisher, and M. Sherer, *Treatment of multiple sleep problems in children with developmental disabilities: faded bedtime with response cost versus bedtime scheduling.* Dev. Med. Child Neurol., 1997. **39**(6): p. 414-418.
45. Quine, L. and K. Wade, *Sleep disturbance in children with severe learning difficulties: an examination and an intervention trial*. 1991: University of Kent at Canterbury, Centre for Research in Health Behaviour.
46. Wiggs, L. and G. Stores, *Behavioural treatment for sleep problems in children with severe learning disabilities and challenging daytime behaviour: effect on sleep patterns of mother and child.* J. Sleep Res., 1998. **7**(2): p. 119-126.
47. Yehuda, S., S. Rabinovitz-Shenkar, and R.L. Carasso, *Effects of essential fatty acids in iron deficient and sleep-disturbed attention deficit hyperactivity disorder (ADHD) children.* Eur. J. Clin. Nutr., 2011. **65**(10): p. 1167-1169.
48. Yu, H.-B. and Y.-Y. Hong, *Acupuncture combined with ear point taping and pressing for sleep disturbance in 30 children with mental retardation.* World Journal of Acupuncture - Moxibustion, 2012. **22**(4): p. 9-12.
49. Markovich, A.N., M.A. Gendron, and P.V. Corkum, *Validating the Children's Sleep Habits Questionnaire against polysomnography and actigraphy in school-aged children.* Frontiers in Psychiatry, 2015. **5**(188).
50. Meltzer, L., et al., *Use of actigraphy for assessment in pediatric sleep research.* Sleep Med Rev, 2012. **16**(5): p. 463-475.
51. Evans, R., J. Scourfield, and S. Murphy, *The unintended consequences of targeting: young people's lived experiences of social and emotional learning interventions.* British Educational Research Journal, 2015. **41**(3): p. 381-397.
52. Divon, S.A. and C.E. Bergstrøm, *Unintended consequences of development interventions: a case of diarrhoeal diseases, Ruhiira, Uganda.* Development in Practice, 2012.
53. Thompson, G., et al., *Unintended consequences of incentive provision for behaviour change and maintenance around childbirth.* PLOS ONE, 2014. **9**(10): p. e111322.
54. Miyar, J. and C.E. Adams, *Content and quality of 10 000 controlled trials in schizophrenia over 60 years.* Schizophrenia Bulletin, 2013. **39**(1): p. 226-229.
55. McDaid, C., et al., *Systematic review of the evidence on orthotic devices for the management of knee instability related to neuromuscular and central nervous system disorders.* BMJ Open, 2017. **7**(e015927).
56. Morris, C., et al., *Informing the NHS Outcomes framework: evaluating meaningful health outcomes for children with neurodisability using multiple methods including systematic review, qualitative research, Delphi survey and consensus meeting.* Health Services and Delivery research, 2014. **2**(15).
57. Macleod, M.R., et al., *Biomedical research: increasing value, reducing waste.* Lancet, 2014. **383**: p. 101-104.
58. Davies, K., et al., *Parental experience of sleep-dirordered breathing in infants with cleft palate: comparing parental and clinical priorities.* The Cleft Palate-Craniofacial Journal, 2018. https://doi.org/10.1177/1055665618770196.
59. Alkhaffaf, B., et al., *Standardising the reporting of outcomes in gastric cancer surgery trials: protocol for the development of a core outcome set and accompanying outcome measurement set (the GASTROS study).* Trials, 2017. **18**(370).
60. Prinsen, C.A.C., et al., *How to select outcome measurement instruments for outcomes included in a "Core Outcome Set"- a practical guideline.* Trials, 2016. **17**(449).
61. Calvert, M., et al., *Reporting of Patient-Reported Outcome Measures in Randomized Trials: The CONSORT PRO Extension.* JAMA, 2013. **309**(8): p. 814-822.

For peer review only - http://bmjopen.bmj.com/site/about/guidelines.xhtml

PRISMA 2009 Checklist

Section/topic	#	Checklist item	Reported on page #
**TITLE**			
Title	1	Identify the report as a systematic review, meta-analysis, or both.	1
**ABSTRACT**			
Structured summary	2	Provide a structured summary including, as applicable: background; objectives; data sources; study eligibility criteria, participants, and interventions; study appraisal and synthesis methods; results; limitations; conclusions and implications of key findings; systematic review registration number.	2
**INTRODUCTION**			
Rationale	3	Describe the rationale for the review in the context of what is already known.	4
Objectives	4	Provide an explicit statement of questions being addressed with reference to participants, interventions, comparisons, outcomes, and study design (PICOS).	4
**METHODS**			
Protocol and registration	5	Indicate if a review protocol exists, if and where it can be accessed (e.g., Web address), and, if available, provide registration information including registration number.	5
Eligibility criteria	6	Specify study characteristics (e.g., PICOS, length of follow-up) and report characteristics (e.g., years considered, language, publication status) used as criteria for eligibility, giving rationale.	5
Information sources	7	Describe all information sources (e.g., databases with dates of coverage, contact with study authors to identify additional studies) in the search and date last searched.	5
Search	8	Present full electronic search strategy for at least one database, including any limits used, such that it could be repeated.	Will be available in HTA report
Study selection	9	State the process for selecting studies (i.e., screening, eligibility, included in systematic review, and, if applicable, included in the meta-analysis).	5
Data collection process	10	Describe method of data extraction from reports (e.g., piloted forms, independently, in duplicate) and any processes for obtaining and confirming data from investigators.	6
Data items	11	List and define all variables for which data were sought (e.g., PICOS, funding sources) and any assumptions and simplifications made.	6
Risk of bias in individual studies	12	Describe methods used for assessing risk of bias of individual studies (including specification of whether this was done at the study or outcome level), and how this information is to be used in any data synthesis.	Will be available in HTA

For peer review only

PRISMA 2009 Checklist

			report not the focus of this paper
Summary measures	13	State the principal summary measures (e.g., risk ratio, difference in means).	n/a
Synthesis of results	14	Describe the methods of handling data and combining results of studies, if done, including measures of consistency (e.g., I^2) for each meta-analysis.	n/a

Section/topic	#	Checklist item	Reported on page #
Risk of bias across studies	15	Specify any assessment of risk of bias that may affect the cumulative evidence (e.g., publication bias, selective reporting within studies).	n/a
Additional analyses	16	Describe methods of additional analyses (e.g., sensitivity or subgroup analyses, meta-regression), if done, indicating which were pre-specified.	n/a
**RESULTS**			
Study selection	17	Give numbers of studies screened, assessed for eligibility, and included in the review, with reasons for exclusions at each stage, ideally with a flow diagram.	6
Study characteristics	18	For each study, present characteristics for which data were extracted (e.g., study size, PICOS, follow-up period) and provide the citations.	11
Risk of bias within studies	19	Present data on risk of bias of each study and, if available, any outcome level assessment (see item 12).	n/a
Results of individual studies	20	For all outcomes considered (benefits or harms), present, for each study: (a) simple summary data for each intervention group (b) effect estimates and confidence intervals, ideally with a forest plot.	n/a
Synthesis of results	21	Present results of each meta-analysis done, including confidence intervals and measures of consistency.	n/a
Risk of bias across studies	22	Present results of any assessment of risk of bias across studies (see Item 15).	n/a
Additional analysis	23	Give results of additional analyses, if done (e.g., sensitivity or subgroup analyses, meta-regression [see Item 16]).	n/a
**DISCUSSION**			
Summary of evidence	24	Summarize the main findings including the strength of evidence for each main outcome; consider their relevance to key groups (e.g., healthcare providers, users, and policy makers).	20-22
Limitations	25	Discuss limitations at study and outcome level (e.g., risk of bias), and at review-level (e.g., incomplete retrieval of identified research, reporting bias).	22
Conclusions	26	Provide a general interpretation of the results in the context of other evidence, and implications for future research.	23

For peer review only

For peer review only - http://bmjopen.bmj.com/site/about/guidelines.xhtml

PRISMA 2009 Checklist

**FUNDING**			
Funding	27	Describe sources of funding for the systematic review and other support (e.g., supply of data); role of funders for the systematic review.	23

From: Moher D, Liberati A, Tetzlaff J, Altman DG, The PRISMA Group (2009). Preferred Reporting Items for Systematic Reviews and Meta-Analyses: The PRISMA Statement. PLoS Med 6(7): e1000097. doi:10.1371/journal.pmed1000097

For more information, visit: **www.prisma-statement.org**.

For peer review only

For peer review only - http://bmjopen.bmj.com/site/about/guidelines.xhtml

BMJ Open

Outcome domains and outcome measures used in studies assessing the effectiveness of interventions to manage non-respiratory sleep disturbances in children with neurodisabilities: a systematic review

Journal:	*BMJ Open*
Manuscript ID	bmjopen-2018-027205.R1
Article Type:	Research
Date Submitted by the Author:	25-Feb-2019
Complete List of Authors:	McDaid, Catriona; University of York, York Trials Unit, Department of Health Sciences<br>Parker, Adwoa; University of York, York Trials Unit, Department of Health Sciences<br>Scantlebury, Arabella; University of York, York Trials Unit, Department of Health Sciences<br>Fairhurst, Caroline; University of York, York Trials Unit, Department of Health Sciences<br>Dawson, Vicky; The Children's Sleep Charity<br>Elphick, Heather; Sheffield Children's NHS Foundation Trust, Department of Respiratory Medicine<br>Hewitt, Catherine; University of York, York Trials Unit, Department of Health Sciences<br>Spiers, Gemma; Newcastle University,<br>Thomas, Megan; Blackpool Teaching Hospitals NHS Foundation Trust<br>Beresford, Bryony; University of York, Social Policy Research Unit
**Primary Subject Heading**:	Paediatrics
Secondary Subject Heading:	Research methods
Keywords:	Developmental neurology & neurodisability < PAEDIATRICS, SLEEP MEDICINE, Clinical trials < THERAPEUTICS

SCHOLARONE™
Manuscripts

Outcome domains and outcome measures used in studies assessing the effectiveness of interventions to manage non-respiratory sleep disturbances in children with neurodisabilities: a systematic review

Catriona McDaid[1], Adwoa Parker[1], Arabella Scantlebury[2], Caroline Fairhurst[1], Vicky Dawson[3], Heather Elphick[4], Catherine Hewitt[1], Gemma Spiers,[2] Megan Thomas[5], Bryony Beresford[6]

[1] York Trials Unit, Department of Health Sciences, University of York, York, UK

[2] Institute for Health and Society, Baddiley-Clark Building, Newcastle Upon-Tyne, UK

[3] The Children's Sleep Charity, Kale Lode, Woodfield Park, Tickhill Road, Doncaster, UK

[4] Sheffield Children's NHS Foundation Trust, Sheffield, UK

[5] Blackpool Teaching Hospitals NHS Foundation Trust, Blackpool, UK

[6]Social Policy Research Unit, University of York, York, UK

Corresponding author: Dr Catriona McDaid, catriona.mcdaid@york.ac.uk

For peer review only

ABSTRACT

Objectives: To assess whether a core outcome set is required for studies evaluating the effectiveness of interventions for non-respiratory sleep disturbances in children with neurodisabilities.

Design: Survey of outcome measures used in primary studies identified by a systematic review.

Data sources: ASSIA; CENTRAL; Cochrane Database of Systematic Reviews; Conference Proceedings Citation Index; CINAHL; DARE; Embase; HMIC; MEDLINE; MEDLINE In-Process; PsycINFO; Science Citation Index; Social Care Online; Social Policy & Practice; ClinicalTrials.gov; WHO International Clinical Trials Registry Platform (ICTRP); and the UK Clinical Trials Gateway were searched up to February 2017.

Eligibility criteria: Studies evaluating pharmacological or non-pharmacological interventions for children (≤ 18 years old) with a neurodisability and experiencing non-respiratory sleep disturbance.

Data extraction and synthesis: Outcome measures were listed from each study and categorised into domains.

Results: Thirty-nine studies assessed five core outcome areas: child sleep, other child outcomes, parent outcomes, adverse events and process measures. There were 54 different measures of child sleep across five domains: global measures; sleep initiation; maintenance; scheduling; and other outcomes. Fifteen non-pharmacological (58%) and four pharmacological studies (31%) reported child outcomes other than sleep using 29 different measures.

One pharmacological and 14 non-pharmacological (54%) studies reported parent outcomes (17 different measures). Eleven melatonin studies (85%) recorded adverse events, with variation in how data were collected and reported. One non-pharmacological study reported an explicit method of collecting on adverse events. Several process measures were reported, related to adherence, feasibility of delivery, acceptability and experiences of receiving the intervention.

Conclusions: There is a lack of consistency between studies in the outcome measures used to assess the effectiveness of interventions for non-respiratory sleep disturbances in children with neurodisabilities. A minimum core outcome set, with international consensus, should be developed in consultation with parents, children and young people, and those involved in supporting families.

Registration number systematic review PROSPERO (CRD42016034067)

Strengths and limitations of this study

For peer review only - http://bmjopen.bmj.com/site/about/guidelines.xhtml

- Includes multiple interventions and populations thus providing a comprehensive overview of outcome measurement in this field.
- Provides data to inform the first stage of development of a core outcome set.
- The study provides efficient use of data from an existing review but some outcomes that were outside the scope of our effectiveness review may have been missed.

For peer review only

For peer review only - http://bmjopen.bmj.com/site/about/guidelines.xhtml

INTRODUCTION

Child sleep problems are associated with poorer educational outcomes and daytime behaviour difficulties[1] as well as having a negative impact on parents, such as heightened levels of parental stress and irritability.[2] Sleep disturbance can impact on all members of the family and it is often the parents' own poor sleep quality which leads to them seeking help with their child's sleep. [3] Sleep disturbances are more common and severe in children with neurodisabilities compared with typically developing children.[4, 5] A recent national research prioritisation exercise ranked management of sleep disturbance for this group of children in the top ten research priorities.[6]

In response to a commissioned call from the UK National Institute for Health Research (NIHR), we undertook a systematic review, to evaluate the effectiveness of pharmacological and non-pharmacological interventions for children with neurodisabilities experiencing non-respiratory sleep disturbances. The NIHR commissioning brief requested a broad systematic review to 'take stock' of the current available evidence in order to inform future research.

One of the challenges we faced while undertaking the review was the diversity of outcomes assessed across the included studies, possibly a reflection of the fact that there is no agreed set of outcomes recommended for use in trials assessing the effectiveness of interventions in this field of research. Based on searches of the COMET database [http://www.comet-initiative.org/ (accessed 21 June 2017)] there is no core outcome set in relation to children with neurodisabilities or typically developing children who experience non-respiratory sleep disturbances. A core outcome set is an agreed minimum, standardised set of outcomes that should be measured and reported in randomised controlled trials (RCTs) for a specific clinical area.[7] They are required to maximise comparability across studies in a specific field in order to facilitate quantitative and narrative synthesis, reduce selective outcome reporting in studies of effectiveness and increase the relevance of results from primary studies and systematic reviews. [7] An important first step, if there is not a core outcome set in a field, is to establish whether one is needed. [8] The breadth of our review, covering both pharmacological and non-pharmacological interventions provided an opportunity to explore whether a core outcome set is required.

The results of the systematic review of effectiveness are available separately (www.journalslibrary.nihr.ac.uk/programmes/hta/1421202/#/).[9] [10] [11] The aim of this paper is to summarise the outcome measures used in the RCTs and other effectiveness studies evaluating pharmacological and non-pharmacological interventions for the management of non-respiratory sleep disturbances in children with neurodisabilities and make recommendations about whether a core outcome set is required.

For peer review only - http://bmjopen.bmj.com/site/about/guidelines.xhtml

METHODS

We used the included studies from our systematic review of treatment effectiveness, which was based on a prospectively registered review protocol (PROSPERO, registration number: CRD42016034067). A summary of the methods relevant to this paper is provided below...

Databases searched

We searched the following without language restrictions up to February 2017: Applied Social Science Abstracts & Indexes (ASSIA); The Cochrane Central Register of Controlled Trials (CENTRAL); Cochrane Database of Systematic Reviews (CDSR); Conference Proceedings Citation Index; Cumulative Index to Nursing & Allied Health (CINAHL); Database of Abstracts of Reviews of Effects (DARE); Embase; Health Management Information Consortium (HMIC); MEDLINE; MEDLINE In-Process; PsycINFO; Science Citation Index; Social Care Online; Social Policy & Practice; ClinicalTrials.gov; WHO International Clinical Trials Registry Platform (ICTRP); and the UK Clinical Trials Gateway. The search strategy for ASSIA is in Supplementary file 1. The search results were loaded into EndNote bibliographic software (version 17.0.2.7390, Thomas Reuters, CO, USA).

Study selection

One researcher screened titles only to remove obviously irrelevant records (10% checked by a second researcher); two researchers then independently screened abstracts and ordered potentially relevant papers; the full papers were screened independently by two researchers. We included studies of children and young people (≤ 18 years old) with neurodisability, experiencing non-respiratory sleep disturbances, which evaluated pharmacological (melatonin, clonidine, and antihistamines) or non-pharmacological Interventions (e.g. behavioural, cognitive-behavioural, self-help resources, complementary therapies). We used the Morris *et al.* definition of neurodisability as "congenital or acquired long-term conditions that are attributed to impairment of the brain and/or neuromuscular system and create functional limitations". [12] A best available evidence approach was used with non-randomised controlled studies and before and after studies included where RCT evidence was not available.

Outcomes

A broad range of outcomes were of interest:

Primary outcomes: child sleep-related outcomes (including parent/carer and child reported outcomes (e.g. using sleep diaries and actigraphy); parent sleep-related outcomes (e.g. quality of sleep); and measures of perceived parenting confidence, efficacy, or understanding of sleep/sleep

For peer review only - http://bmjopen.bmj.com/site/about/guidelines.xhtml

management (which can be a specific focus of parent training/behavioural interventions which seek to change how parents manage sleep disturbance).

Secondary outcomes: child-related quality of life, daytime behaviour and cognition; parent/carer outcomes such as quality of life and well-being, physical well-being, mental well-being; family functioning; and adverse events.

Data extraction and synthesis

For each study the specific outcome measures used were extracted with details of how the outcome was defined or described by the author (where reported) and, where relevant, the method of assessment e.g. child/parent reported outcome measure, sleep diaries, actigraphy, polysomnography. Data were extracted by one researcher and checked by a second.

Outcomes were tabulated and grouped into core areas, then by outcome domain and specific outcome measure used. This was undertaken by a single researcher and discussed with the wider team. The data were described in a narrative synthesis grouped by the categories.

Patient and Public Involvement

There was patient and public involvement in the decision by NIHR to commission the overall project focusing on sleep disturbances in children with neurodisabilities. Three parents of children with neurodisabilities, identified from an existing parent consultation group agreed to be involved with the project. They were invited to project team meetings, which took place three times during the project, and were consulted via email regarding the implications of the review findings. Their contributions provided useful contextual information. In addition, a member of a sleep charity, which supports parents, was involved at all stages of the project and is a co-author (VD) of the publication.

RESULTS

Overview of included studies

After removal of duplicates 15,745 records were screened and 387 full-text records assessed for eligibility. Thirty-nine studies (reported across 64 articles) were included: 13 studies of melatonin, published between 1996 and 2012 and 26 studies of parent-directed, environmental, dietary and complementary medicine interventions, published between 1991 and 2016 (Table 1). The latter group of studies are referred to as non-pharmacological for convenience. The studies were most commonly undertaken in the UK (36%) and USA (26%) followed by Australia (15%) and Canada (13%). Single studies were reported from the Netherlands, Italy, Israel and China. Study designs

For peer review only - http://bmjopen.bmj.com/site/about/guidelines.xhtml

encompassed RCTs (both parallel-group and crossover), and, for the non-pharmacological interventions, controlled before and after studies, and one-arm before and after studies were also included.

The studies assessed five core outcome areas: child sleep, other child outcomes, parent-related, adverse events and process outcomes. All of the studies (n=39) reported at least one child sleep-related outcome: 19 (49%) reported other types of child outcomes such as daytime behaviour and cognition; and 14 reported parent/carer outcomes (36%) (Table 1).

For the non-pharmacological studies, the first outcome measurement time points ranged from immediately post intervention to two months post intervention and five measured additional follow-up time points. All the melatonin studies had a single follow-up time point immediately following the completion of the intervention, which ranged from 10 days to 12 weeks post randomisation.

Child sleep outcomes

Table 2 reports the child sleep outcome measures used by at least one study. Fifty-four different measures of child related sleep outcomes were reported. There were five domains: global assessments of sleep (n=17 outcome measures); sleep initiation (n=10), sleep maintenance (n=18), sleep scheduling (n=4) and other outcomes (n=5).

In the melatonin studies, total sleep time (TST) was the most commonly reported global sleep outcome, reported by 12 of the 13 included studies (92%). Four reported TST derived from parent-completed sleep diaries and actigraphy; [13] [14] [15] [16] three reported actigraphy derived data only; [17] [18] [19] six sleep diary derived data. [20] [21] [22] [23] [24] [25] The most common way to measure the sleep initiation domain was sleep onset latency (SOL, 85%); and within the sleep maintenance domain was night awakening (46%). As with TST, these outcomes were assessed using both actigraphy and sleep diary (reporting results of both or actigraphy only) or using sleep diary only. Sleep scheduling and parent-reported global measures of sleep were rarely reported in the melatonin studies (Table 2).

For the non-pharmacological studies, the sleep outcome measures used were more disparate with only one measure reported by more than half the studies. The most commonly reported global measure of sleep (and the most commonly reported overall) was the parent-reported Child Sleep Habits Questionnaire (CSHQ; 58%). TST calculated from actigraphy or parent-reported sleep diary data was much less commonly used (42%) compared to melatonin studies. Six studies reported actigraphy derived data, [26] [27] [28] 27] [29] [30] three reported parent completed sleep diary derived data, [31] [32] [33] one reported actigraphy and diary derived data,[34] and one reported data based on “semiweekly” phone calls to parents.[35] For the sleep initiation domain, the most

For peer review only - http://bmjopen.bmj.com/site/about/guidelines.xhtml

commonly reported was SOL (39%); for sleep maintenance it was number of night awakenings (15%) and wake after sleep onset (19%); sleep scheduling was rarely reported. There was some overlap between pharmacological and non-pharmacological studies in the measures used, most commonly TST, sleep efficiency and SOL (Table 2).

Even where studies were measuring the same outcome, there was variation in how this was measured, though often the method used was not specified. For example, in terms of wake time, one study used actigraphy and defined the outcome as the last epoch of actigraphically assessed immobility before the start of a 10 minute consecutive period of activity around the time of leaving bed;[18] two used parent-reported wake time using a sleep diary (no further definition provided);[16, 36] and one study used a momentary time sampling observation of children who were in-patients.[37] Wake after sleep onset (WASO) and night awakening both assess sleep maintenance, but different approaches to measurement were used. One study used actigraphy to measure night awakenings (definition not provided);[38] one used a sleep diary (defining night awakenings as the number of awakenings per week that parents were aware of);[33] and the other two did not provide a definition.[35, 36] All studies reporting WASO used actigraphy, two of which defined WASO as a sum of the all wake epochs during the sleep period (total time awake during the night excluding the sleep onset latency period)[28, 39] (two did not specify how it was defined[27, 29]). Where SOL was defined, there was also some variation. Definitions included time from lights out to sleep onset, the time from start of bedtime routine to sleep, the amount of time between when the child was put to be and they fell asleep, and the time between taking melatonin and falling asleep.

Other child outcomes

Nineteen studies reported child outcomes other than sleep (Table 3): 15 non-pharmacological studies (58%) and four pharmacological studies (31%). The domains assessed were child behaviour, quality of life, ADHD symptoms, cognition, school-related, and other. Twenty-nine different measures were used across the 18 studies and no single measure was used by more than three of the studies, though most by only one or two studies (Table 3). The measures used ranged from validated tools to study-specific questionnaires.

It was not always clear whether studies had used the same version of a measure. For example, there are two versions of the Daily Parent Rating of Evening and Morning Behaviour (DREMB) scale; [40] the original version (DREMB) and a revised version (DREMB-R) with an item on irritability removed. Both studies using this outcome measure referenced the same source for the scale suggesting they

For peer review only - http://bmjopen.bmj.com/site/about/guidelines.xhtml

used the same version. However, this could not be confirmed as only one gave details of the score range of the version used.[27, 41]

Finally, we encountered a lack of clarity in reporting with respect to whether sub-scale and/or total scores were used. Thus, for the two studies which used the Child Behaviour Checklist, one reported the eight subscales scores plus a total score and daytime internalising behaviour problem score (calculated from scores on three of the subscales), [42] the other study presented insufficient information about the scores used. [39]

Adverse outcomes

Eleven melatonin studies (85%) recorded adverse events, with variation across studies in how data were collected and reported. Different approaches were described to measuring adverse events, with varying levels of standardisation, including: a standardised assessment tool which classified events into seven domains;[13] elicitation of adverse events during in-person/phone-call visits by the study team;[16, 17] using study specific questionnaires completed by parents;[22] [23] one reported using a standardised form;[15] open-ended interviews;[14, 18] one reported by parents using an unspecified method;[25] and in one study it was unclear how the data were collected.[19] One non-pharmacological study reported a specific method of collecting on adverse events: making a 24 hour telephone line available to parents for reporting adverse events and gathering the information at weekly parents reviews (face-to-face or telephone). [38] While other non-pharmacological studies reported some adverse events it was unclear whether there was a systematic method of gathering this information.

Parent outcomes

Fourteen non-pharmacological studies (54%) reported parent outcomes (Table 4). A range of outcome domains were assessed, and measures used were not consistent. Domains included: parental mental health (three outcome measures across 5 studies), parenting (for example, perceived confidence, efficacy or knowledge in relation to managing their child's sleep problem) (five outcome measures across 9 studies), quality of parental sleep (six outcome measures across 3 studies) and a range of other outcomes including attendance at work and locus of control (three measures across four studies). One melatonin study assessed parent outcomes; specifically, it used the Epworth Sleepiness Scale to assess parental daytime sleepiness.[13]

Process outcomes

Several other outcomes and process measures were reported; related to adherence, feasibility of delivery, acceptability and experiences of receiving the intervention. These included: adherence to

For peer review only - http://bmjopen.bmj.com/site/about/guidelines.xhtml

treatment (based on medication count (pharmacological studies only), intervention sessions attended, adherence to specific components of the intervention; withdrawal from study); various study-specific individual items or questionnaires to ascertain parents views about specific aspects of the intervention or overall views, for example, the proportion of participants rating treatment on a five point rating scale from “too tough” to “too soft”; rating of overall treatment helpfulness (visual analogue scale) and specific components of the intervention; study specific Caregivers Acceptance of Treatment Survey (Likert Scale response format); a modified version of the Program Evaluation Questionnaires and qualitative interviews. There was variation in the extent to which this information was reported in a standardised way, for example, limited detail in the methods used.

For peer review only

Table 1 Summary of included studies

Study	Year Country	Type of neurodisability	Intervention	Study design	Child outcomes Sleep	Child outcomes Other	Parent outcomes	Adverse events
**MELATONIN**								
**Appleton *et al.* [13]**	2012 UK	DD alone or with other condition	Melatonin	Parallel RCT	X	X	X	X
**Camfield *et al.*[20]**	1996 Canada	Mixed	Melatonin	N of 1 crossover RCT	X			
**Cortesi *et al.*[17]**	2012 Italy	ASD	Melatonin	Parallel RCT	X	X		X
**der Heijden *et al.*[18]**	2007 Netherlands	ADHD	Melatonin	Parallel RCT	X	X		X
**Dodge and Wilson [23]**	2001 USA	Mixed	Melatonin	Crossover RCT	X			X
**Garstang and Wallis [21]**	2006 UK	ASD with or without LD	Melatonin	Crossover RCT	X			
**Hancock *et al.* [25]**	2005 UK	Tuberous sclerosis	Melatonin	Crossover RCT	X			X
**Jain *et al.*[16]**	2015 USA	Epilepsy	Melatonin	Crossover RCT	X	X		X
**Jan *et al.*[24]**	2000 Canada	Mixed	Melatonin	Crossover RCT	X			X
**Wasdell *et al.* [14]**	2008 Canada	Mixed	Melatonin	Crossover RCT	X			X
**Weiss *et al.*[15]**	2006 Canada	ADHD	Melatonin	Crossover RCT	x	X		X
**Wirojanan *et al.*[19]**	2009 USA	Mixed	Melatonin	Crossover RCT	X			X

For peer review only - http://bmjopen.bmj.com/site/about/guidelines.xhtml

Study	Year Country	Type of neurodisability	Intervention	Study design	Child outcomes Sleep	Child outcomes Other	Parent outcomes	Adverse events
**Wright *et al*.[22]**	2011 UK	Mixed	Melatonin	Crossover RCT	X			X
			**NON-PHARMACOLOGICAL**					
**Adkins *et al*.[28]**	2012 USA	Mixed	Parent-directed, non-tailored	Parallel RCT	X			
**Austin *et al*.[36]**	2013 Australia	Mixed	Parent-directed, tailored	Before-after	X	X		
**Beresford *et al*.[3]**	2012 UK	Mixed	Parent-directed, tailored	Parallel RCT	X		X	
**Beresford, *et al*.[3], Intervention 2**	2012 UK	Mixed	Parent-directed, tailored	Before-after	X		X	
**Beresford *et al*.[3] Intervention 3**	2012 UK	Mixed	Parent-directed non-tailored	Before-after	X		X	
**Beresford *et al*.[3] Intervention 4**	2012 UK	Mixed	Parent-directed non-tailored	Before-after	X		X	
**Bramble, (1997) [43]**	1997 UK	Mixed	Parent-directed non-tailored	Before-after	X	X	X	
**Francis and Dempster[31]**	2002 Australia	Mixed	Valerian	Crossover RCT	X	X		
**Gringras, *et al*.[34]**	2014 UK	Mixed	Weighted blanket	Crossover RCT	X	X		X
**Guilleminault, *et al*.[32]**	1993 USA	LD	Light therapy+behavioural programme	Before-after	X			
**Hiscock *et al*.[27]**	2015 Australia	ADHD plus LD or ASD	Parent-directed, tailored	Parallel RCT	X	X	X	
**Johnson, *et al*.[26]**	2013 USA	Autism & ASD	Parent-directed, tailored	Parallel RCT	X			
**Malow *et al*.[39]**	2014 USA	Mixed	Parent-directed non-tailored	Parallel RCT	X	X		

For peer review only - http://bmjopen.bmj.com/site/about/guidelines.xhtml

Study	Year<br>Country	Type of neurodisability	Intervention	Study design	Child outcomes<br>Sleep	 Other	Parent outcomes	Adverse events
**Montgomery et al.[44]**	2004<br>UK	Mixed	Parent-directed non-tailored	Parallel RCT	X			
**Moss *et al.*[45]**	2014<br>Australia	Mixed	Parent-directed, tailored	Parallel RCT	X	X	X	
**Oriel *et al.*[35]**	2016<br>USA	ASD	Aquatic exercise programme	A-B-A	X			
**Peppers *et al.*[46]**	2016<br>USA	NR	Non-comprehensive, parent-directed	Before-after	X	X	X	
**Piazza *et al.*[47]**	1997<br>USA	Mixed	Behavioural	Parallel RCT	X			
**Quine and Wade[48]**	1991<br>UK	LD	Parent-directed, tailored	Before-after	X	X	X	
**Reed *et al.*[29]**	2009<br>Canada	ASD	Parent-directed non-tailored	Before-after	X	X	X	
**Sciberras *et al.*[41]**	2011<br>Australia	ADHD	Parent-directed, tailored	Parallel RCT	X	X	X	
**Weiskop, *et al.*[33]**	2005<br>Australia	Mixed	Parent-directed, tailored	Before-after	X			
**Wiggs and Stores[30]**	1998<br>UK	Mixed	Non-comprehensive, parent-directed	RCT	X	X	X	
**Yehuda *et al.*[49]**	2011<br>Israel	ADHD	Essential fatty acids	Controlled before-after	X	X		X
**Yu *et al.*[42]**	2015<br>Hong Kong	ASD & Asperger syndrome	Parent-directed non-tailored	Before-after	X	X	X	
**Yu and Hong[50]**	2012<br>China	LD	Acupuncture and earpoint taping	Before-after	X			

ADHD attention deficit hyperactivity disorder; ASD autism spectrum disorder; DD developmental delay; LD learning disability; NR not reported

For peer review only

For peer review only - http://bmjopen.bmj.com/site/about/guidelines.xhtml

[a] definitions for how studies were classified as parent directed and tailored/non-tailored are available in the effectiveness review report (www.journalslibrary.nihr.ac.uk/programmes/hta/1421202/#/)

Table 2 Child Sleep related outcomes

		Melatonin(n=13)		Non-pharmacological (n=26)		All studies (n=39)
	**Sleep outcome**	**Number of studies (%)**	**Studies measuring outcome**	**Number of studies (%)**	**Studies measuring outcome**	**Number of studies (%)**
Global measures	Total sleep time	12 (92)	[13-23, 25]	11 (42)	[26-33, 35, 38, 39]	23 (59)
	Sleep efficiency	5 (38)	[13, 14, 16-18]	5 (19)	[26-28, 38, 39]	10 (26)
	Longest sleep episode	1 (8)	[14]	0 (0)		1 (3)
	Changes in sleep pattern	1 (8)	[24]	0 (0)		1 (3)
	CSHQ	1 (8)	[17]	15 (58)	[3, 36][a, b, c,d] [27, 29, 35, 38, 39, 41, 42, 45, 46, 50]	16 (41)
	SBQ	1 (8)	[16]	0 (0)		1 (3)
	Parent-set Child Sleep Goals	0 (0)		4 (15)	[3] [a, b, c, d]	4 (10)
	Composite Sleep Index (from modified version of Simonds and Parraga Sleep Questionnaire)	0 (0)		3 (12)	[26, 30, 38]	3 (8)
	Goal Attainment Scale	0 (0)		2 (8)	[33, 45]	2 (5)
	Change in goal attainment rating	0 (0)		1 (4)	[3]	1 (3)
	Sleep problems over past 4 weeks	0 (0)		1 (4)	[27]	1 (3)

For peer review only - http://bmjopen.bmj.com/site/about/guidelines.xhtml

		Melatonin(n=13)		Non-pharmacological (n=26)		All studies (n=39)
	**Sleep outcome**	**Number of studies (%)**	**Studies measuring outcome**	**Number of studies (%)**	**Studies measuring outcome**	**Number of studies (%)**
	Family Inventory of Sleep Habits	0 (0)		3 (12)	[29, 39, 42]	3 (8)
	Composite Sleep Disturbance Score	0 (0)		1 (4)	[44]	1 (3)
	Sleep quality (visual analogue scale)	0 (0)		1 (4)	[31]	1 (3)
	Sleep improvement – child and parent rating scale	0 (0)		1 (4)	[38]	1 (3)
	Quality of sleep – child smiley face rating	0 (0)		1 (4)	[38]	1 (3)
	Quality of sleep – Likert rating scale	0 (0)		1 (4)	[49]	1 (3)
Sleep initiation	Sleep onset latency	11 (85)	[13-19, 21-23, 25]	10 (39)	[26, 28, 29, 31, 33, 35, 36, 38, 39, 43]	21 (54)
	Bedtime	2 (15)	[16, 17]	1 (4)	[29]	3 (8)
	Sleep onset	2 (15)	[18, 19]	1 (4)	[37]	3 (8)
	Bedtime resistance	0 (0)		1 (4)	[36]	1 (3)
	Bedtime routine	0 (0)		1 (4)	[36]	1 (3)
	Number of pre-sleep disturbances	0 (0)		1 (4)	[33]	1 (3)
	Falling asleep in own bed	0 (0)		1 (4)	[33]	1 (3)
	Difficulty falling asleep (single item Likert scale)	1 (8)	[18]	0 (0)		1 (3)
	Severity of bedtime settling problems (Behaviour Screening Questionnaire)			1(4)	[48]	1 (3)
	Time to settle at night	0 (0)		2 (8)	[33, 48]	2 (5)

For peer review only - http://bmjopen.bmj.com/site/about/guidelines.xhtml

		Melatonin(n=13)		Non-pharmacological (n=26)		All studies (n=39)
	**Sleep outcome**	**Number of studies (%)**	**Studies measuring outcome**	**Number of studies (%)**	**Studies measuring outcome**	**Number of studies (%)**
Sleep maintenance	Night awakenings	6 (46)	[14, 19, 20, 22, 23, 25]	5 (19)	[33, 35, 36, 38, 47]	11 (28)
	Number of nights without waking	1 (8)	[20]	0 (0)		1 (3)
	Wake time	2 (15)	[16, 18]	2 (8)	[36, 37]	4 (10)
	Time spent moving during sleep period	1 (15)	[18]	0 (0)		1 (3)
	Co-sleeping	0 (0)		2 (8)	[33, 36]	2 (5)
	Number of nights not sleeping in own bed			1 (4)	[48]	1 (3)
	Wake After Sleep Onset	2 (15)	[16, 17]	4 (15)	[27-29, 39]	6 (15)
	Severity of sleep problem (VAS) (incorporating disturbed sleep)	0 (0)		1 (4)	[43]	1 (3)
	Movement during sleep	0 (0)		1 (4)	[30]	1 (3)
	Movement index	0 (0)		1 (4)	[30]	1 (3)
	Fragmentation index	0 (0)		1 (4)	[30]	1 (3)
	Time spent awake during night	0 (0)		3 (12)	[31, 38, 48]	3 (8)
	Proportion of nights with > 1 wakening	0 (0)		1 (4)	[38]	1 (3)
	Parent/caregiver reported ‘no’ or ‘mild’ sleep problems	0 (0%)		1 (4)	[41]	1 (2.6%)
	Longest wake and sleep periods during 24 hour cycle	0 (0)		1 (4)	[32]	1 (3)
	Time of night wakes and return to sleep	0 (0)		1 (4)	[37]	1 (3)
	Hours of disturbed sleep	0 (0)		1 (4)	[37]	1 (3)
	Sleep Disturbance Index	0 (0)		1 (4)	[36]	1 (3)

	Sleep outcome	Melatonin(n=13) Number of studies (%)	Studies measuring outcome	Non-pharmacological (n=26) Number of studies (%)	Studies measuring outcome	All studies (n=39) Number of studies (%)
Sleep scheduling	Naptime	1 (8)	[17]	0 (0)		1 (3)
	Napping	0 (0)		1 (4)	[36]	1 (3)
	Distribution of sleep bouts during 24 hour cycle	0 (0)		1 (4)	[32]	1 (3)
	Degree of fatigue during the day – Likert rating scale	0 (0)		1 (4)	[49]	1 (3)
Other	Interdaily stability	1 (8)	[18]	0 (0)		1 (3)
	Interdaily variability	1 (8)	[18]	0 (0)		1 (3)
	L5	1 (8)	[18]	0 (0)		1 (3)
	Arousal Index (AASM)	1 (8)	[16]	0 (0)		1 (3)
	Percentage of time in each sleep stage	1 (8)	[16]	0 (0)		1 (3)

AASM Arousal Index; CSHQ Children's Sleep Habits Questionnaire; SBQ Sleep Behaviour Questionnaire; VAS visual analogue scale [a] Beresford, Intervention 1, [b] Beresford Intervention 2, [c] Beresford Intervention 3

Table 3 Other child outcomes

	Outcome	Melatonin (n=13) Number of studies (%)	Studies measuring outcome	Non-pharmacological (n=26) Number of studies (%)	Studies measuring outcome	All (n=39) Number of studies (%)
Behaviour	Daily Parent Rating of Evening and Morning Behaviour Scale	0 (0)		2 (8)	[27, 41]	2 (5)

	Outcome	Melatonin (n=13) Number of studies (%)	Melatonin (n=13) Studies measuring outcome	Non-pharmacological (n=26) Number of studies (%)	Non-pharmacological (n=26) Studies measuring outcome	All (n=39) Number of studies (%)
	Aberrant Behaviour Checklist	1 (8)	[13]	2 (8)	[30, 38]	3 (8)
	Behaviour Problem Index	0 (0)		2 (8)	[43, 48]	2 (5)
	Child Behaviour Checklist	1 (8)	[18]	2 (8)	[39, 42]	3 (8)
	Repetitive Behaviour Scale – Revised	0 (0)0		2 (8)	[29, 39]	2 (5)
	Child daytime behaviour (based on diary)	0 (0)		1 (4)	[31]	1 (3)
	Developmental Behaviour Checklist-Parent Version	0 (0)		2 (8)	[36, 45]	2 (5)
	Sensory Behaviour Questionnaire (unpublished)	0 (0)		1 (4)	[38]	1 (3)
	Behaviour Assessment System for Children-Parent Rating Scale	1 (8)	[16]	0 (0)		1 (3)
Quality of life	Paediatric Quality of Life Inventory 4.0	1 (8)	[13]	3 (12)	[27, 39, 41]	4 (10)
	Quality of Life in Childhood Epilepsy	1 (8)	[16]	0 (0)		1 (3)
	Level of good mood in general (Likert scale)	1 (8)	[15]	1 (4)	[49]	2 (5)
	TNO-AZL Quality of Life Questionnaire	1 (8)	[18]	0 (0)		1 (3)
ADHD symptoms	Vanderblit ADHD Symptom Checklist	0 (0)		1 (4)	[46]	1 (3)
	ADHD Rating Scale IV	0 (0)		2 (8)	[27, 41]	2 (5)
	Conner's Attention Deficit Scale-Parent Version	1 (8)	[15]	0 (0)		1 (3)

For peer review only - http://bmjopen.bmj.com/site/about/guidelines.xhtml

	Outcome	Melatonin (n=13) Number of studies (%)	Melatonin (n=13) Studies measuring outcome	Non-pharmacological (n=26) Number of studies (%)	Non-pharmacological (n=26) Studies measuring outcome	All (n=39) Number of studies (%)
Cognitive	Ability to concentrate during the day, mainly at school (Likert scale)	0 (0)		1 (4)	[49]	1 (3)
	Working memory test battery[a]	0 (0)		1 (4)	[27]	1 (3)
	Sustained attention dots task-completion time	1 (8)	[18]	0 (0)		1 (3)
	Sustained attention dots task-inaccuracy[b]	1 (8)	[18]	0 (0)		1 (3)
	Erikson Task – reaction time and error incidence[c]	1 (8)	[18]	0 (0)		1 (3)
School-related	Homework completion (Likert scale)	0 (0)		1 (4)	[49]	1 (3)
	School attendance	0 (0)		2 (8)	[27, 41]	2 (5)
	Teacher's Report Form	1 (8)	[18]	0 (0)		1 (3)
Other	Meal times	0 (0)		1 (4)	[36]	1 (3)
	Parent report of other professional help sought	0 (0)		1 (4)	[27]	1 (3)
	Parental Concerns Questionnaire	0 (0)		1 (4)	[29, 42]	1 (3)
	Strength and Difficulties Questionnaire	0 (0)		1 (4)	[27]	1 (3)
	Parent and teacher reported core problems[d]	1 (8)	[18]	0 (0)		1 (3)

[a]Backwards digit recall, counting recall, listening recall; [b]Percentage of misses plus false alarms relative to the total number of trials; [c]Reaction time and error incidence between congruent and incongruent tasks; [d]Core problems were spontaneously and individual defined by parents

For peer review only - http://bmjopen.bmj.com/site/about/guidelines.xhtml

Table 4 Parent/carer outcomes (non-pharmacological studies n=26)

Domain	Outcome Measure	Number of studies (%)	Studies measuring outcome
Parental mental health and well-being	Depression Anxiety Stress Scales	2 (8)	[27, 41]
	The Parenting Stress Index-Short Form	3 (12)	[29, 42, 45]
	The Malaise Inventory	3 (12)	[30, 43, 48]
Parenting (e.g. Perceived confidence efficacy or knowledge)	Perceived ability to control own and partner's sleep difficulties (visual analogue scale)	1 (4)	[30]
	Parental satisfaction with their ability to cope with their child's sleep (Likert scale)	1 (4)	[30]
	Parent satisfaction with child's sleep	1 (4)	[30]
	Parenting Sense of Competence scale (plus satisfaction and efficacy subscales)	5 (19)	[3][a,b,c,d] [39]
	Knowledge of Behavioural Principles as Applied to Children	1 (4)	[48]
Parental sleep	Maternal Sleep Scale	1 (4)	[43]
	Pittsburgh Sleep Quality Index	1 (4)	[42]
	Maternal total sleep time (actigraphy)	1 (4)	[30]
	Other actigraphy measures of sleep (sleep period, activity score, movement index, fragmentation index)	1 (4)	[30]
	Epworth Sleepiness Scale	1 (4)	[30]
	Parental satisfaction with own sleep (Likert scale)	1 (4)	[30]
Other	Work attendance (number of days missed or late for work)	2 (8)	[27, 41]
	Parent Satisfaction Likert Survey	1 (4)	[46]
	Parental locus of Control	1 (4)	[30]

For peer review only - http://bmjopen.bmj.com/site/about/guidelines.xhtml

DISCUSSION

Principal findings

The findings suggest a strong imperative to develop a core outcome set to be used in trials in this field. Over 70 different outcome measures were reported across 39 studies, the vast majority of measures reported by three or fewer studies. This greatly impedes analysis of data in systematic reviews due to lack of comparability across studies. It also makes it difficult to assess the likelihood of selective outcome reporting, with the associated risks of overestimating intervention effectiveness. It is unlikely that all these measures have equal importance for children and parents, leading to uncertainty when making judgements about the relevance of specific study results.

There were five core areas assessed in the included studies: child sleep (six domains); other child outcomes (five domains); parent outcomes (four domains) adverse events and process measures. This suggests some consensus at the broadest level about what is relevant to measure; however, as the classification became more granular the diversity increased.

There was overlap between pharmacological and non-pharmacological studies in terms of the child sleep domains assessed and, to some extent, the measures used, reflecting the fact that, regardless of mechanism, a key target of both types of intervention was some aspect of child sleep. However, compared to the melatonin studies, there was more focus in the non-pharmacological studies on the core areas of child non-sleep outcomes, parent outcomes and family experience. This reflects the complex nature of these interventions, with parent understanding and perceived competency in managing their child's sleep being implicit in the mechanism of action. The parent outcome domains were mental health, parenting (perceived confidence, efficacy, or knowledge) and quality of sleep. Arguably, it is surprising that more studies of parent-directed interventions did not assess impact on parental perceived competence/confidence and that melatonin studies have not taken as family-centred approach to assessing the effects of the intervention in this population.

The most widely used outcome measure in the melatonin studies was total sleep time (measured using actigraphy and/or sleep diary) and in the non-pharmacological studies was the CSHQ. Although they are both within the core domain of child sleep and provide a global assessment, they differ considerably in level of objectivity, aspects of sleep assessed, feasibility and cost.[51] They could be viewed as complementary measures to be used together, though only six studies used both. Even amongst these more common outcome measures there was variation between studies in how they were used. Lack of standard scoring rules and variation in definitions used for actigraphy-derived outcomes has been identified as an area of concern, which is supported by our research. [52]

For peer review only

For peer review only - http://bmjopen.bmj.com/site/about/guidelines.xhtml

Adverse events were reported in the majority of melatonin trials but rarely considered in the non-pharmacological studies. Standardisation of the reporting and data collection methods for adverse events in future trials is important to understand the safety of pharmacological interventions. This also has relevance for non-pharmacological studies as well, where interventions may have unintended consequences. [53-55]

The diversity of outcome measures used significantly limited both the narrative and quantitative syntheses conducted for the review of the effectiveness of these interventions.[9] Our findings are consistent with the results of studies in other fields exploring the extent of consistency of outcome measures used and in other evidence syntheses.[56, 57]

Unanswered questions and future research

A minimum core outcome set for use in effectiveness studies would greatly assist the usefulness of research in this field. This need not restrict researchers using outcome measures outside the core outcome set, where relevant to the specific study.[58] An agreed minimum core outcome set would improve the ability to make comparisons between studies and ensure that the outcomes being used in studies are of relevance to children, their parents and also the healthcare professionals and others involved in providing support. Although sleep has been identified as an important outcome by young people with neurodisabilities and their parents it remains unclear what aspects of sleep are most important to them, for example whether it is the child's total amount of sleep time, when they sleep, how many times they wake during the night etc. The importance to them of improving other outcomes such as child daytime behaviour or parental outcomes as part of sleep management interventions and which should be prioritised is also unclear.[59]

Effectiveness research that does not assess relevant and important outcomes is a source of avoidable waste.[60] The core outcome set should be developed in consultation with parents and carers, children themselves where possible, and healthcare professionals and others involved in supporting parents and children, using a structured process such as that developed by the COMET Group.[8] It is currently unclear whether the outcomes being assessed in studies evaluating sleep disturbance interventions are those that are important to families, both in terms of children's sleep outcomes and parents' sleep outcomes. Research in other fields has shown a disparity between the outcomes important to patients and those that are assessed in intervention studies. [57, 61, 62] Development of a core outcome set should be international in scope to ensure future widespread adoption. In addition to identifying core outcomes, consensus will be required regarding appropriate measurement instruments. This should draw on appropriate methods to ensure selection of reliable

For peer review only - http://bmjopen.bmj.com/site/about/guidelines.xhtml

and valid measures such as those used by the COSMIN initiative.[63] Factors such as feasibility and respondent burden will also be important to consider. It will also be necessary to draw on existing comparative evidence on how objective and subjective sleep outcome measures perform, and the extent to which they are capturing unique dimensions of sleep.[64, 65] Whilst child-report measures exist, work on their psychometric properties is limited and none appear to have been developed for children with any significant degree of learning difficulty. [66] The heterogeneity in outcome measures used by studies in our review was compounded by the variation across studies in the clarity of information provided on how some outcomes were measured. Future research should follow appropriate reporting guidelines.[67]

A key decision point at the outset would be whether a core outcome set should be developed separately for pharmacological and non-pharmacological interventions. We suggest that an appropriate approach would be to have a minimum core outcome set common across interventions with additional outcomes agreed where relevant for the two types of interventions. For parent-directed interventions, children's sleep outcomes are mediated by parent outcomes achieved by the intervention (for example, parental acquisition of new knowledge and understanding of sleep, training in managing sleep disturbance). Formalising and refining a theory of change for such interventions would support and inform the development of a core outcome set. This is relevant for both child and parent outcomes. Additionally, consideration is required of the variation across different types of neurodisability, where there may be desirable outcomes for one group not shared by other groups. The alternatives are to develop separate core outcome sets for children with sleep disturbance for each neurodisability or to have an overall consensus building exercise that prioritises a minimum core outcome set across conditions, with additional condition-specific outcomes identified. The latter is likely to be more feasible from a resource perspective.

Consistency in follow-up time points across studies of similar interventions, at least at the primary follow-up point, is required in order to support comparisons between studies in future systematic reviews and meta-analyses. This will require reaching consensus with regard to the most clinically meaningful follow-up time points, and whether evaluations should also seek to investigate maintenance of outcomes. Again, for non-pharmacological studies in particular, this would benefit from being based on an evidence-informed theory of change as the implementation of newly acquired knowledge and skills in managing a child's sleep may take time to have an effect. The final follow-up time-point for most studies in our effectiveness review only allowed consideration of short-term outcomes. Future studies need to consider what longer-term follow-ups should be incorporated into study designs.

Strengths and weaknesses of the study

For peer review only - http://bmjopen.bmj.com/site/about/guidelines.xhtml

The systematic review included a wide range of neurodisabilities and interventions in children aged 18 years or under. It therefore provides a comprehensive overview of the outcome domains assessed in this field of research and the outcome measures being used. However, we may have under-estimated the range of outcomes being assessed as this work was undertaken as part of a systematic review of effectiveness. Thus, although the outcomes of interest were very broad we did not exhaustively include all outcomes; therefore, we may have failed to identify some outcomes, for example some physiological aspects of sleep. In addition, we assigned the measures to one of a number of outcome domains, others doing the same task may allocate differently or attribute to different domains. However, these factors are unlikely to change the conclusions. The description of outcome domains and measures used provides important evidence for the field in moving towards developing a core outcome set. While these will contribute to the development of a list of outcomes for a Delphi survey, as a first stage of developing a core outcome set, it is important that this is supplemented by the views of parents and children, healthcare professionals involved in designing and delivering interventions and researchers working in the field.

CONCLUSION

There is a lack of consistency between studies in the outcome measures used to assess the effectiveness of interventions for non-respiratory sleep disturbances in children with neurodisabilities. This hampers evidence synthesis and creates uncertainty about the relevance of study findings to parents and children. A minimum core outcome set, with international consensus, should be developed in consultation with parents, children and young people, and those involved in supporting families.

FUNDING STATEMENT

This work is derived from a project funded by the National Institute for Health Research (NIHR) HTA Programme (project number 14/212/02). Further information is available at https://www.journalslibrary.nihr.ac.uk/programmes/hta/1421202/#/

This report presents independent research commissioned by the NIHR. The views and opinions expressed by authors in this publication are those of the authors and do not necessarily reflect those of the NHS, the NIHR, MRC, CCF, NETSCC, the HTA Programme or the Department of Health

For peer review only - http://bmjopen.bmj.com/site/about/guidelines.xhtml

COMPETING INTERESTS

Bryony Beresford and Megan Thomas were authors of studies included in the review, but were not involved in data extraction or quality assessment of these studies. Catriona McDaid is a member of the NIHR HTA & EME Editorial Board. Catherine Hewitt is a member of HTA Commissioning Board.

ACKNOWLEDGEMENTS

We would like to thank our parent advisors for their interest and enthusiasm for the project and their contributions at project meetings.

AUTHORS CONTRIBUTIONS

Catriona McDaid: jointly responsible for writing the protocol and had shared responsibility for co-ordinating and leading the project; provided advice and input to all elements of the project. Wrote sections of main report and wrote and revised drafts of this paper

Adwoa Parker and Arabella Scantlebury: equally contributed to study selection, data extraction, quality assessment, and synthesis and report writing of the main report. Commented on drafts of this paper.

Caroline Fairhurst: conducted the data analysis, contributed to the report writing and commented on drafts of this paper.

Catherine Hewitt: contributed to the protocol, provided methodological advice throughout the project, commented on drafts of the main report and drafts of this paper.

Gemma Spiers: worked on the project February – September 2016. During that time she was responsible for the day-to-day running of the project and led on screening, retrieval, data extraction and quality appraisal. Commented on drafts of this paper.

Vicki Dawson, Heather Elphick, Megan Thomas: provided expert clinical advice throughout the project, contributed to screening and data extraction processes and to drafts of the report, commented on drafts of this paper.

Bryony Beresford: jointly responsible for writing the review protocol and had shared responsibility for co-ordinating and leading the project; provided advice and input to all elements of the project. Wrote sections of main report and commented on drafts of this paper.

DATA SHARING STATEMENT

For peer review only - http://bmjopen.bmj.com/site/about/guidelines.xhtml

The data were derived from published studies and are publicly available in the main body and appendices of the HTA Monograph.

For peer review only

For peer review only - http://bmjopen.bmj.com/site/about/guidelines.xhtml

REFERENCES

1. Simola, P., *Sleep problems and their implications from preschool to school age*. Studies in Educational SCiences. 2014: Käyttäytymistieteiden laitos (Helsingin yliopisto).
2. Doo, S. and Y.K. Wing, *Sleep problems of children with pervasive developmental disorders: correlation with parental stress.* Developmental medicine and child neurology, 2006. **48**(8): p. 650-655.
3. Beresford, B., et al., *Interventions E: Individually Delivered Sleep Management Interventions*, in *Managing behaviour and sleep problems in disabled children: An investigation into the effectiveness and costs of parent-training interventions*. 2012, University of Kent: London.
4. Tietze, A.L., et al., *Sleep disturbances in children with multiple disabilities.* Sleep Med Rev, 2012. **16**(2): p. 117-27.
5. Dorris, L., et al., *Sleep problems in children with neurological disorders.* Dev Neurorehabil, 2008. **11**(2): p. 95-114.
6. Morris, C., et al., *Setting research priorities to improve the health of children and young people with neurodisability: a British Academy of Childhood Disability-James Lind Alliance Research Priority Setting Partnership.* BMJ open, 2015. **5**(1): p. e006233.
7. Williamson, P.R., et al., *Developing core outcome sets for clinical trials: issues to consider.* Trials, 2012. **13**(132).
8. Williamson, P.R., et al., *The COMET Handbook: version 1.0.* Trials, 2017. **18**(3): p. 280.
9. Scantlebury, A., et al., *Non-pharmacological interventions for non-respiratory sleep disturbance in children with neurodisabilities: a systematic review.* Developmental Medicine & Child Neurology, 2018.
10. Beresford, B., et al., *Pharmacological and non-pharmacological interventions for non-respiratory sleep disturbance in children with neurodisabilities: a systematic review.* Health Technol Assess, 2018. **22**(60): p. 1-296.
11. Parker, A., et al., *Oral melatonin for non-respiratory sleep disturbance in children with neurodisabilities: systematic review and meta-analysis.* Developmental Medicine and Child Neurology, 2019. **doi: 10.1111/dmcn.14157. [Epub ahead of print]**.
12. Morris, C., et al., *Towards a definition of neurodisability: a Delphi survey.* Dev Med Child Neurol, 2013. **55**(12): p. 1103-8.
13. Appleton, R.E., et al., *The use of MElatonin in children with neurodevelopmental disorders and impaired sleep: a randomised, double-blind, placebo-controlled, parallel study (MENDS).* Health Technol. Assess., 2012. **16**(40): p. i-239.
14. Wasdell, M.B., et al., *A randomized, placebo-controlled trial of controlled release melatonin treatment of delayed sleep phase syndrome and impaired sleep maintenance in children with neurodevelopmental disabilities.* J. Pineal Res., 2008. **44**(1): p. 57-64.
15. Weiss, M.D., et al., *Sleep hygiene and melatonin treatment for children and adolescents with ADHD and initial insomnia.* J. Am. Acad. Child Adolesc. Psychiatry, 2006. **45**(5): p. 512-519.
16. Jain, S.V., et al., *Melatonin improves sleep in children with epilepsy: a randomized, double-blind, crossover study.* Sleep Med., 2015. **16**(5): p. 637-644.
17. Cortesi, F., et al., *Controlled-release melatonin, singly and combined with cognitive behavioural therapy, for persistent insomnia in children with autism spectrum disorders: a randomized placebo-controlled trial.* J. Sleep Res., 2012. **21**(6): p. 700-709.
18. der Heijden, K.B.V.A.N., et al., *Effect of melatonin on sleep, behavior, and cognition in ADHD and chronic sleep-onset insomnia.* J. Am. Acad. Child Adolesc. Psychiatry, 2007. **46**(2): p. 233-241.
19. Wirojanan, J., et al., *The efficacy of melatonin for sleep problems in children with autism, fragile X syndrome, or autism and fragile X syndrome.[Erratum appears in J Clin Sleep Med. 2010 Aug 15;6(4):preceding 311].* J. Clin. Sleep Med., 2009. **5**(2): p. 145-150.
20. Camfield, P., et al., *Melatonin appears ineffective in children with intellectual deficits and fragmented sleep: six" N of 1" trials.* J. Child Neurol., 1996. **11**(4): p. 341-343.

For peer review only - http://bmjopen.bmj.com/site/about/guidelines.xhtml

21. Garstang, J. and M. Wallis, *Randomized controlled trial of melatonin for children with autistic spectrum disorders and sleep problems.* Child Care Health Dev., 2006. **32**(5): p. 585-589.
22. Wright, B., et al., *Melatonin versus placebo in children with autism spectrum conditions and severe sleep problems not amenable to behaviour management strategies: a randomised controlled crossover trial.* J. Autism Dev. Disord., 2011. **41**(2): p. 175-184.
23. Dodge, N.N. and G.A. Wilson, *Melatonin for treatment of sleep disorders in children with developmental disabilities.* J. Child Neurol., 2001. **16**(8): p. 581-584.
24. Jan, J.E., et al., *Clinical trials of controlled-release melatonin in children with sleep--wake cycle disorders.* J. Pineal Res., 2000. **29**(1): p. 34-39.
25. Hancock, E., F. O'Callaghan, and J.P. Osborne, *Effect of melatonin dosage on sleep disorder in tuberous sclerosis complex.* J. Child Neurol., 2005. **20**(1): p. 78-80.
26. Johnson, C.R., et al., *Behavioral parent training to address sleep disturbances in young children with autism spectrum disorder: a pilot trial.* Sleep Med., 2013. **14**(10): p. 995-1004.
27. Hiscock, H., et al., *Impact of a behavioral sleep intervention on ADHD symptoms, child sleep and parent health: a randomized controlled trial.* BMJ, 2015. **350**: p. h68.
28. Adkins, K.W., et al., *Effects of a standardized pamphlet on insomnia in children with autism spectrum disorders.* Pediatrics, 2012. **130 Suppl 2**: p. S139-44.
29. Reed, H.E., et al., *Parent-based sleep education workshops in autism.* J. Child Neurol., 2009. **24**(8): p. 936-945.
30. Wiggs, L. and G. Stores, *Behavioural treatment for sleep problems in children with severe learning disabilities and challenging daytime behaviour: effect on sleep patterns of mother and child.* J. Sleep Res., 1998. **7**(2): p. 119-126.
31. Francis, A.J.P. and R.J.W. Dempster, *Effect of valerian, Valeriana edulis, on sleep difficulties in children with intellectual deficits: randomised trial.* Phytomedicine, 2002. **9**(4): p. 273-279.
32. Guilleminault, C., et al., *Light therapy as treatment of dyschronosis in brain impaired children.* Eur. J. Pediatr., 1993. **152**(9): p. 754-759.
33. Weiskop, S., A. Richdale, and J. Matthews, *Behavioural treatment to reduce sleep problems in children with autism or fragile X syndrome.* Dev. Med. Child Neurol., 2005. **47**(2): p. 94-104.
34. Gringras, P., et al., *Weighted Blankets and Sleep in Autistic Children—A Randomized Controlled Trial.* Pediatrics, 2014. **134**(2): p. 298-306.
35. Oriel, K.N., et al., *The Impact of Aquatic Exercise on Sleep Behaviors in Children With Autism Spectrum Disorder.* Focus on Autism & Other Developmental Disabilities, 2016. **31**(4): p. 254-261.
36. Austin, K., J.E. Gordon, and A. O'Connell, *Preliminary Evaluation of Sleepwise Program for Children With Sleep Disturbance and Developmental Delay.* Child Fam. Behav. Ther., 2013. **35**(3): p. 195-211.
37. Piazza, C.C. and W. Fisher, *A faded bedtime with response cost protocol for treatment of multiple sleep problems in children.* J. Appl. Behav. Anal., 1991. **24**(1): p. 129-140.
38. Gringras, P., et al., *Weighted blankets and sleep in autistic children - a randomised controlled trial.* J. Sleep Res., 2014. **23**(Suppl.): p. 320-320.
39. Malow, B.A., et al., *Parent-based sleep education for children with autism spectrum disorders.* J. Autism Dev. Disord., 2014. **44**(1): p. 216-228.
40. Faraone, S.V., A. Childress, and S.B. Wigal, *Reliability and validity of the Daily Parent Rating of Evening and Morning Behaviour Scale, Revised.* Journal of Attention Disorders, 2015.
41. Sciberras, E., et al., *Managing sleep problems in school aged children with ADHD: a pilot randomised controlled trial.* Sleep Med., 2011. **12**(9): p. 932-935.
42. Yu, X.T., et al., *Extended Parent-based Behavioural Education Improves Sleep in Children with Autism Spectrum Disorder.* HK J Paediatr (New Series), 2015. **20**(4): p. 219-225.
43. Bramble, D., *Rapid-acting treatment for a common sleep problem.* Developmental Medicine & Child Neurology, 1997. **39**(8): p. 543-547.

For peer review only

44. Montgomery, P., G. Stores, and L. Wiggs, *The relative efficacy of two brief treatments for sleep problems in young learning disabled (mentally retarded) children: a randomised controlled trial.* Arch. Dis. Child., 2004. **89**(2): p. 125-130.
45. Moss, A.H.B., J.E. Gordon, and A. O'Connell, *Impact of sleepwise: an intervention for youth with developmental disabilities and sleep disturbance.* J. Autism Dev. Disord., 2014. **44**(7): p. 1695-1707.
46. Peppers, K.H., et al., *An Intervention to Promote Sleep and Reduce ADHD Symptoms.* Journal of Pediatric Health Care, 2016. **30**(6): p. e43-e48.
47. Piazza, C.C., W.W. Fisher, and M. Sherer, *Treatment of multiple sleep problems in children with developmental disabilities: faded bedtime with response cost versus bedtime scheduling.* Dev. Med. Child Neurol., 1997. **39**(6): p. 414-418.
48. Quine, L. and K. Wade, *Sleep disturbance in children with severe learning difficulties: an examination and an intervention trial*. 1991: University of Kent at Canterbury, Centre for Research in Health Behaviour.
49. Yehuda, S., S. Rabinovitz-Shenkar, and R.L. Carasso, *Effects of essential fatty acids in iron deficient and sleep-disturbed attention deficit hyperactivity disorder (ADHD) children.* Eur. J. Clin. Nutr., 2011. **65**(10): p. 1167-1169.
50. Yu, H.-B. and Y.-Y. Hong, *Acupuncture combined with ear point taping and pressing for sleep disturbance in 30 children with mental retardation.* World Journal of Acupuncture - Moxibustion, 2012. **22**(4): p. 9-12.
51. Markovich, A.N., M.A. Gendron, and P.V. Corkum, *Validating the Children's Sleep Habits Questionnaire against polysomnography and actigraphy in school-aged children.* Frontiers in Psychiatry, 2015. **5**(188).
52. Meltzer, L., et al., *Use of actigraphy for assessment in pediatric sleep research.* Sleep Med Rev, 2012. **16**(5): p. 463-475.
53. Evans, R., J. Scourfield, and S. Murphy, *The unintended consequences of targeting: young people's lived experiences of social and emotional learning interventions.* British Educational Research Journal, 2015. **41**(3): p. 381-397.
54. Divon, S.A. and C.E. Bergstrøm, *Unintended consequences of development interventions: a case of diarrhoeal diseases, Ruhiira, Uganda.* Development in Practice, 2012.
55. Thompson, G., et al., *Unintended consequences of incentive provision for behaviour change and maintenance around childbirth.* PLOS ONE, 2014. **9**(10): p. e111322.
56. Miyar, J. and C.E. Adams, *Content and quality of 10 000 controlled trials in schizophrenia over 60 years.* Schizophrenia Bulletin, 2013. **39**(1): p. 226-229.
57. McDaid, C., et al., *Systematic review of the evidence on orthotic devices for the management of knee instability related to neuromuscular and central nervous system disorders.* BMJ Open, 2017. **7**(e015927).
58. Boers, M., et al., *Developing core outcome measurement sets for clinical trials: OMERACT Filter 2.0.* Journal of Clinical Epidemiology, 2014. **67**: p. 745-753.
59. Morris, C., et al., *Informing the NHS Outcomes framework: evaluating meaningful health outcomes for children with neurodisability using multiple methods including systematic review, qualitative research, Delphi survey and consensus meeting.* Health Services and Delivery research, 2014. **2**(15).
60. Macleod, M.R., et al., *Biomedical research: increasing value, reducing waste.* Lancet, 2014. **383**: p. 101-104.
61. Davies, K., et al., *Parental experience of sleep-dirordered breathing in infants with cleft palate: comparing parental and clinical priorities.* The Cleft Palate-Craniofacial Journal, 2018. https://doi.org/10.1177/1055665618770196.
62. Alkhaffaf, B., et al., *Standardising the reporting of outcomes in gastric cancer surgery trials: protocol for the development of a core outcome set and accompanying outcome measurement set (the GASTROS study).* Trials, 2017. **18**(370).

For peer review only - http://bmjopen.bmj.com/site/about/guidelines.xhtml

63. Prinsen, C.A.C., et al., *How to select outcome measurement instruments for outcomes included in a "Core Outcome Set"- a practical guideline.* Trials, 2016. **17**(449).
64. Bauer, K. and S. Blunden, *How accurate is subjective reporting of childhood sleep patterns? A review of the literature and implications for practice.* Current Pediatric Reviews, 2008. **4**(2): p. 134-142.
65. Smith, M., et al., *Use of actigraphy for the evaluation of sleep disorders and circadian rhythm sleep-wake disorders: an American Academy of Sleep Medicine systematic review, meta-analysis, and GRADE assessment.* J Clin Sleep Med, 2018. **14**(7): p. 1209-1230.
66. Erwin, A. and L. Bashore, *Subjective sleep measures in children: Self-report.* Frontiers in Pediatrics, 2017. **5**(22): p. doi: 10.3389/fped.2017.00022.
67. Calvert, M., et al., *Reporting of Patient-Reported Outcome Measures in Randomized Trials: The CONSORT PRO Extension.* JAMA, 2013. **309**(8): p. 814-822.

For peer review only

SEARCH STRATEGY

Applied Social Science Abstracts & Indexes (ASSIA) Via ProQuest

((((SU.EXACT("Sleep disorders") OR SU.EXACT("Sleep problems")) OR SU.EXACT("Narcolepsy")) OR (("bed time*" NEAR/3 (dysfunction* OR disorder* OR difficult* OR disrupt* OR disturb* OR delay* OR problem*)) OR (bedtime* NEAR/3 (dysfunction* OR disorder* OR difficult* OR disrupt* OR disturb* OR delay* OR problem*)) OR ("bed time*" NEAR/3 (initial* OR pattern* OR routine* OR practice* OR maintain* OR intervention* OR schedule*)) OR ("bed time*" NEAR/3 (initial* OR pattern* OR routine* OR practice* OR maintain* OR intervention* OR schedule*))) OR ((night* NEAR/3 (dysfunction* OR disorder* OR difficult* OR disrupt* OR disturb* OR delay* OR problem*)) OR (night* NEAR/3 (settle* OR settling OR wake* OR awake OR wakeful* OR waking* OR awaking* OR awakening* OR wakening*)) OR (nocturnal NEAR/3 (dysfunction* OR disorder* OR difficult* OR disrupt* OR disturb* OR delay* OR problem*)) OR (nocturnal NEAR/3 (settle* OR settling OR wake* OR awake OR wakeful* OR waking* OR awaking* OR awakening* OR wakening*))) OR ((sleep* NEAR/3 (dysfunction* OR disorder* OR difficult* OR disrupt* OR disturb* OR delay* OR problem*)) OR (sleep* NEAR/3 (settle* OR settling OR wake* OR awake OR wakeful* OR waking* OR awaking* OR awakening* OR wakening*)) OR (sleep* NEAR/3 (initial* OR pattern* OR routine* OR practice* OR maintain* OR intervention* OR schedule*)) OR (sleepless* OR insomnia* OR parasomnia* OR "night terror*" OR nightterror* OR "night mare*" OR nightmare*) OR ("sleepwalk*" OR "nighthawk*" .) OR (sleepwalk* OR sleepwalk* OR "sleep walk*" OR somnambulism) OR (narcolepsy OR "nocturnal hyperkinesia"))) AND ((SU.EXACT("Children") OR SU.EXACT("adolescentce") OR SU.EXACT("Infants")) OR (adolescent* OR baby OR babies OR child OR children OR boy OR boys OR girl OR girls OR infant* OR infancy* OR juvenile* OR paediatric OR pediatric OR preschooler* OR schoolboy* OR schoolgirl* OR schoolchild* OR teens OR teenager* OR toddler* OR youth OR youths OR "young people" OR "young person*"))) AND (((SU.EXACT("Developmentally disabled children") OR SU.EXACT("Developmentally delayed children")) OR (SU.EXACT("Developmental delays") OR SU.EXACT("Developmental disorders")) OR SU.EXACT("Angelman syndrome") OR (SU.EXACT("Attention deficit disorder") OR SU.EXACT("Attention deficit hyperactivity disorder")) OR SU.EXACT("Conduct disorders") OR SU.EXACT("Complex partial seizure disorder" OR "Epilepsy" OR "Idiopathic childhood epilepsy" OR "Landau-Kleffner syndrome" OR "Panayiotopoulos syndrome" OR "Temporal lobe epilepsy") OR

For peer review only - http://bmjopen.bmj.com/site/about/guidelines.xhtml

SU.EXACT("Cerebral palsy") OR SU.EXACT("Down's syndrome") OR SU.EXACT("Fragile X syndrome") OR (SU.EXACT("Prader - Willi syndrome") OR SU.EXACT("Prader-Willi syndrome"))) OR (SU.EXACT("Rett syndrome") OR SU.EXACT("Smith-Magenis syndrome") OR (SU.EXACT("Williams-Beuren syndrome") OR SU.EXACT("Williams' syndrome")) OR (ADHD or "attention deficit" OR "angelman syndrome") OR (autism or autistic or asperges* OR "cerebral palsy") OR ("conduct disorder*" OR epilepsy or epileptic) OR ("Down* syndrome" OR "Fragile x syndrome") OR ("Prader Willi Syndrome" OR "Prader-Willi Syndrome") OR ("Rett syndrome" OR "Williams syndrome")) OR (developmental NEAR/2 (disability* OR delay*)) OR neurodisability* OR (neurodevelopment* NEAR/3 (delay* OR disability* OR disease* OR disorder* OR dysfunction)) OR (neuromotor* NEAR/3 (delay* OR disability* OR disease* OR disorder* OR dysfunction)) OR (neuropsychiatr* NEAR/3 (delay* OR disability* OR disease* OR disorder* OR dysfunction)) OR (neuropsychol* NEAR/3 (delay* OR disability* OR disease* OR disorder* OR dysfunction)))

PRISMA 2009 Checklist

Section/topic	#	Checklist item	Reported on page #
**TITLE**			
Title	1	Identify the report as a systematic review, meta-analysis, or both.	1
**ABSTRACT**			
Structured summary	2	Provide a structured summary including, as applicable: background; objectives; data sources; study eligibility criteria, participants, and interventions; study appraisal and synthesis methods; results; limitations; conclusions and implications of key findings; systematic review registration number.	2
**INTRODUCTION**			
Rationale	3	Describe the rationale for the review in the context of what is already known.	4
Objectives	4	Provide an explicit statement of questions being addressed with reference to participants, interventions, comparisons, outcomes, and study design (PICOS).	4
**METHODS**			
Protocol and registration	5	Indicate if a review protocol exists, if and where it can be accessed (e.g., Web address), and, if available, provide registration information including registration number.	5
Eligibility criteria	6	Specify study characteristics (e.g., PICOS, length of follow-up) and report characteristics (e.g., years considered, language, publication status) used as criteria for eligibility, giving rationale.	5
Information sources	7	Describe all information sources (e.g., databases with dates of coverage, contact with study authors to identify additional studies) in the search and date last searched.	5
Search	8	Present full electronic search strategy for at least one database, including any limits used, such that it could be repeated.	Will be available in HTA report
Study selection	9	State the process for selecting studies (i.e., screening, eligibility, included in systematic review, and, if applicable, included in the meta-analysis).	5
Data collection process	10	Describe method of data extraction from reports (e.g., piloted forms, independently, in duplicate) and any processes for obtaining and confirming data from investigators.	6
Data items	11	List and define all variables for which data were sought (e.g., PICOS, funding sources) and any assumptions and simplifications made.	6
Risk of bias in individual studies	12	Describe methods used for assessing risk of bias of individual studies (including specification of whether this was done at the study or outcome level), and how this information is to be used in any data synthesis.	Will be available in HTA

For peer review only

PRISMA 2009 Checklist

			report not the focus of this paper
Summary measures	13	State the principal summary measures (e.g., risk ratio, difference in means).	n/a
Synthesis of results	14	Describe the methods of handling data and combining results of studies, if done, including measures of consistency (e.g., I^2) for each meta-analysis.	n/a

Section/topic	#	Checklist item	Reported on page #
Risk of bias across studies	15	Specify any assessment of risk of bias that may affect the cumulative evidence (e.g., publication bias, selective reporting within studies).	n/a
Additional analyses	16	Describe methods of additional analyses (e.g., sensitivity or subgroup analyses, meta-regression), if done, indicating which were pre-specified.	n/a
**RESULTS**			
Study selection	17	Give numbers of studies screened, assessed for eligibility, and included in the review, with reasons for exclusions at each stage, ideally with a flow diagram.	6
Study characteristics	18	For each study, present characteristics for which data were extracted (e.g., study size, PICOS, follow-up period) and provide the citations.	11
Risk of bias within studies	19	Present data on risk of bias of each study and, if available, any outcome level assessment (see item 12).	n/a
Results of individual studies	20	For all outcomes considered (benefits or harms), present, for each study: (a) simple summary data for each intervention group (b) effect estimates and confidence intervals, ideally with a forest plot.	n/a
Synthesis of results	21	Present results of each meta-analysis done, including confidence intervals and measures of consistency.	n/a
Risk of bias across studies	22	Present results of any assessment of risk of bias across studies (see Item 15).	n/a
Additional analysis	23	Give results of additional analyses, if done (e.g., sensitivity or subgroup analyses, meta-regression [see Item 16]).	n/a
**DISCUSSION**			
Summary of evidence	24	Summarize the main findings including the strength of evidence for each main outcome; consider their relevance to key groups (e.g., healthcare providers, users, and policy makers).	20-22
Limitations	25	Discuss limitations at study and outcome level (e.g., risk of bias), and at review-level (e.g., incomplete retrieval of identified research, reporting bias).	22
Conclusions	26	Provide a general interpretation of the results in the context of other evidence, and implications for future research.	23

For peer review only - http://bmjopen.bmj.com/site/about/guidelines.xhtml

PRISMA 2009 Checklist

FUNDING			
Funding	27	Describe sources of funding for the systematic review and other support (e.g., supply of data); role of funders for the systematic review.	23

From: Moher D, Liberati A, Tetzlaff J, Altman DG, The PRISMA Group (2009). Preferred Reporting Items for Systematic Reviews and Meta-Analyses: The PRISMA Statement. PLoS Med 6(7): e1000097. doi:10.1371/journal.pmed1000097

For more information, visit: www.prisma-statement.org.

Page 2 of 2

For peer review only
